# Low‐Thermal‐Budget Doping of 2D Materials in Ambient Air Exemplified by Synthesis of Boron‐Doped Reduced Graphene Oxide

**DOI:** 10.1002/advs.201903318

**Published:** 2020-02-22

**Authors:** Jun‐Hwe Cha, Dong‐Ha Kim, Cheolmin Park, Seon‐Jin Choi, Ji‐Soo Jang, Sang Yoon Yang, Il‐Doo Kim, Sung‐Yool Choi

**Affiliations:** ^1^ School of Electrical Engineering Graphene/2D Materials Research Center Center for Advanced Materials Discovery towards 3D Displays Korea Advanced Institute of Science and Technology (KAIST) 291 Daehak‐ro, Yuseong‐gu Daejeon 34141 Republic of Korea; ^2^ Department of Materials Science and Engineering Korea Advanced Institute of Science and Technology (KAIST) 291 Daehak‐ro, Yuseong‐gu Daejeon 34141 Republic of Korea; ^3^ Division of Materials Science and Engineering Hanyang University Wangsimni‐ro, Seongdong‐gu Seoul 04763 Republic of Korea

**Keywords:** flash irradiation, gas sensors, graphene oxide, low‐thermal‐budget doping

## Abstract

Graphene oxide (GO) doping and reduction allow for physicochemical property modification to suit practical application needs. Herein, the challenge of simultaneous low‐thermal‐budget heteroatom doping of GO and its reduction in ambient air is addressed through the synthesis of B‐doped reduced GO (B@rGO) by flash irradiation of boric acid loaded onto a GO support with intense pulsed light (IPL). The effects of light power and number of shots on the in‐depth sequential doping and reduction mechanisms are investigated by ex situ X‐ray photoelectron spectroscopy and direct millisecond‐scale temperature measurements (temperature >1600 °C, < 10‐millisecond duration, ramping rate of 5.3 × 10^5^ °C s^−1^). Single‐flash IPL allows the large‐scale synthesis of substantially doped B@rGO (≈3.60 at% B) to be realized with a thermal budget 10^6^‐fold lower than that of conventional thermal methods, and the prepared material with abundant B active sites is employed for highly sensitive and selective room‐temperature NO_2_ sensing. Thus, this work showcases the great potential of optical annealing for millisecond‐scale ultrafast reduction and heteroatom doping of GO in ambient air, which allows the tuning of multiple physicochemical GO properties.

## Graphical abstract

Simultaneous heteroatom doping and reduction of graphene oxide (GO) in ambient air by a low‐thermal‐budget process is carried out through single‐shot irradiation using a Xe flash lamp. Compared to pristine reduced GO (rGO), B‐doped rGO features improve NO_2_ sensing performance (better response and reversibility), especially in a highly humid atmosphere.

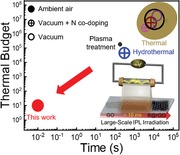

## 1 Introduction

2D materials offer high specific surface area with abundant reaction sites, confined thickness on an atomic scale, and other benefits. Graphene^[^[qv: 1,2]^]^ (as the first 2D material to be discovered) and materials based thereon, which can be cost‐effectively produced on a large scale by direct reduction of graphene oxide (GO),^[^[qv: 3,4]^]^ have been extensively investigated and used in numerous applications. Despite the large specific surface area, high mechanical strength, and ultrahigh carrier mobility of graphene, its practical applicability is limited by the lack of a band gap and catalytic activity,^[^[qv: 5,6]^]^ which has inspired studies on the intrinsic property modification of graphene due to surface modification via heteroatom doping.^[^[qv: 7–10]^]^ In particular, B‐doped graphene exhibits distinct *p*‐type conductivity and a tunable band gap,^[^[qv: 11]^]^ featuring a better NO_2_ sensing performance than pristine graphene^[^[qv: 12,13]^]^ and endowing related materials with promising electrocatalytic and electrochemical properties.^[^[qv: 6,10,14,15]^]^ In this sense, the development of a cost‐effective and mass‐production‐suitable process for simultaneous GO doping and reduction is a task of high importance.

Wang et al. developed a one‐pot hydrothermal synthesis (180 °C, 12 h) of B‐doped reduced GO (B@rGO) and demonstrated the high performance of this material in Na‐ion batteries.^[^[qv: 16]^]^ Putri et al. prepared N‐/B‐co‐doped rGO via 2‐h thermal annealing at 800 °C, showing the great promise of this material for application in the hydrogen evolution reaction.^[^[qv: 17]^]^ Li et al. prepared B@rGO using dielectric barrier discharge plasma with H_2_ as working gas.^[^[qv: 18]^]^ In the aforementioned reports, GO was exposed to a reducing atmosphere and elevated temperature for several hours to achieve reduction and doping. In this sense, the preparation of heteroatom‐doped rGO through a facile and low‐thermal‐budget (LTB) process remains a big challenge, which makes methods for millisecond‐scale ultrafast reduction coupled with heteroatom doping in ambient air highly sought after.

Optical annealing based on the photothermal effect can be induced by two main optical approaches, that is, the monochromatic and broadband light sources. Optical annealing by the photothermal effect offers an attractive and facile way of tuning the properties of the material. Lasers with monochromatic light sources allow the target materials to selectively absorb the wavelength by specific selection of the nanomaterials and their sizes. However, the small spot sizes (≈µm) of lasers can lead to a time‐consuming serial process with low productivity, even with the ultra‐short process time (<ns). On the other hand, lamps with a broadband light spectrum feature relatively large beam sizes (≈cm), offering the advantages of rapid (µs to ms) large‐area processing and high compatibility with the roll‐to‐roll process, thus enabling low‐cost LTB mass production. Besides, the use of lamps for optical treatment enables a variety of materials to be optically treated and have excited electrons, such as phonons, regardless of their electrical band structures, while a laser system has to be adjusted based on the samples to treat.^[^[qv: 19]^]^ Accordingly, optical sintering induced by irradiation with intense pulsed light (IPL) has been widely exploited in the welding of metal nanowires in transparent electrodes, nanostructure growth, and morphology engineering of 2D materials.^[^[qv: 20–22]^]^ Moreover, the reduction of nanomaterials through photothermal effects has been demonstrated for metal nanoparticles and nanowires.^[^[qv: 23–25]^]^ However, to the best of our knowledge, the simultaneous millisecond‐scale doping and reduction of GO by means of optical annealing in ambient air has not been well explored.

Herein, we suggest a facile LTB approach of GO doping and reduction in ambient air, using boric acid (BA) as a B source, GO‐coated glass substrates as supports, and IPL irradiation. The effects of IPL power and number of shots on reduction and doping mechanisms are investigated by direct millisecond‐scale temperature measurements performed using an infrared (IR) sensor system and ex situ X‐ray photoelectron spectroscopy (XPS). The rapid temperature rise upon flash IPL irradiation with a large‐area beam is shown to result in GO doping (≈3.60 at% B) and reduction, inducing a morphology change to a high‐surface‐area open‐pore structure. The room‐temperature NO_2_ sensing performance of the thus obtained B@rGO is demonstrated to exceed those of pristine GO and rGO in terms of both response and reversibility, especially under controlled humidity conditions.

## 2 Results and Discussion

**Figure 1:**
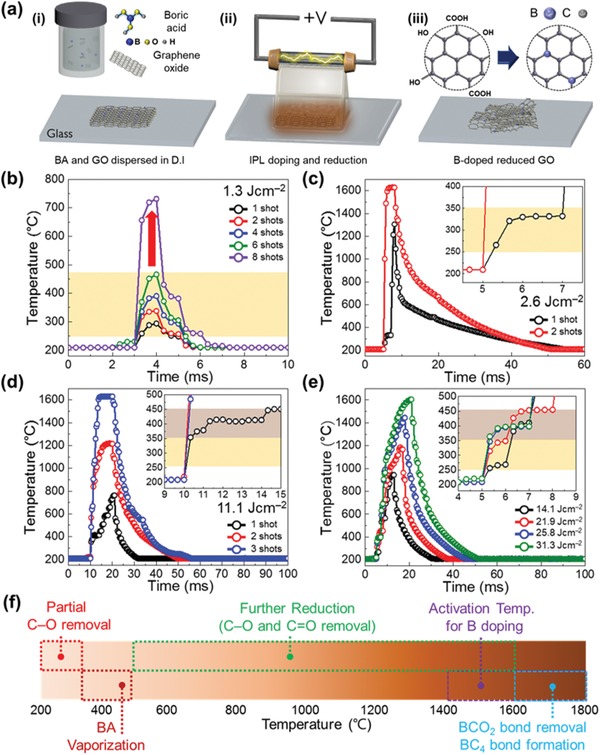
a) Schematic illustration of the photothermal effect–induced simultaneous doping and reduction of BA@GO in ambient air. Temperature–time curves of pristine GO for pulses with flash light energy densities of b) 1.3 and c) 2.6 J cm^−2^; d) corresponding curves of BA@GO obtained at an energy density of 11.1 J cm^−2^. e) Temperature–time curves of BA@GO recorded at a constant applied voltage of 370 V for flash light energy densities of 14.1, 21.9, 25.8, and 31.3 J cm^−2^ corresponding to pulse‐on times of 7, 10, 12, and 15 ms, respectively. f) Temperature‐affected sequential reactions of BA@GO in ambient air induced by the photothermal effect.

**Figure** **1**a illustrates the millisecond‐scale (<10 ms) optical annealing procedures used to prepare B@rGO. BA, known to promote dehydration and additional deoxygenation reactions on the GO surface at a low temperature (Figure S1, Supporting Information),^[^[qv: 26,27]^]^ was uniformly dissolved in an aqueous GO dispersion by ultrasonication, and the obtained solution was drop‐coated on glass substrates (Figure 1a–i) and dried to afford BA crystallites uniformly distributed on pristine GO (BA@GO) (Figure S2, Supporting Information). BA@GO–coated glass substrates were placed on the IPL equipment stage and exposed to a single millisecond‐scale light pulse generated by the Xe lamp (Figure 1a–ii). During irradiation with IPL, light absorption by GO resulted in a large temperature rise due to photothermal effects, which allowed for LTB doping of GO sheets with B as well as their reduction (Figure 1a–iii).

To elucidate the details of millisecond‐scale processes, temperature change was monitored as a function of IPL pulse number using an IR sensor system. Samples were placed 4 cm away from the IPL quartz plate to secure space for IR light passage to the sensor system (Figure S3, Supporting Information). Temperatures measured with an emissivity of 1.00 were corrected using Equation (1):

(1) $$T_{\text{measured}}^{\;-1}\;\;=\;\;T_{\text{corrected}}^{\;-1}\;\;+\;\;\lambda_{\text{detect}}\;\;\times \;\;\ln\left(\varepsilon_{\text{measured}}\text{/}\varepsilon_{\text{corrected}}\right)\times \;\;C_{2}^{\;-1}$$

where *T*_measured_ is the temperature measured by the IR sensor for an IR sensor emissivity setting (ε_measured_) of 1.00, *T*_corrected_ is the temperature corrected by considering the real emissivity of materials (ε_corrected_ = 0.79), λ_detect_ is the IR sensor detection wavelength (2.3 µm), and *C*_2_ = 14388 µm K^−1^ is the second radiant constant.^[^[qv: 28,29]^]^ Besides, the effect of temperature on the progress of GO reduction and doping were probed by ex situ XPS. Note that despite the fact that the emissivity of GO is calculated with the IR sensor and is analogous to the emissivity values of other carbon‐based materials, the emissivity of GO will constantly be changing, depending on the reduction degree and doping conditions, leading to a possible error in the temperature calculation. To investigate the optical reduction behaviors of pristine GO, we studied the effect of IPL energy and number of shots on its temperature profile (Figure 1b,c). Note that the time between each light pulse for multiple shots was longer than 1 s. Given that the heat accumulation effect can only occur when the next pulse is induced within milliseconds, the interval between each pulse is too long for the heat accumulation effect to be utilized. When GO was irradiated at a relatively weak energy density (1.3 J cm^−2^), temperature increased with increasing pulse number (Figure 1b). This behavior was ascribed to the partial reduction of GO and the concomitant increase of its sheet conductivity, which resulted in photothermal effect–induced heating upon exposure of GO semiconducting domains to photons with above‐band‐gap energies.^[^[qv: 30–32]^]^ Given that the band gap of GO lies between 1.0 and 2.2 eV, depending on the reduction degree, light emitted by the Xe flash lamp was preferred to monochromatic (e.g., laser) light for optical GO treatment.^[^[qv: 33,34]^]^ Notably, temperature increased only slowly (from 293.7 to 466.2 °C) as the number of shots increased from one to six, whereas a much more rapid increase was observed from shot seven onward, which suggested that most oxygenated groups were removed, and the reduction process mostly occurred within the range of 250–450 °C. In the case of higher energy density (2.6 J cm^−2^), the temperature increased rapidly and stayed constant at ≈300 °C for 2 ms (Figure 1c). As the reduction process was mostly finished during the first shot, an abrupt increase of temperature to >1630 °C was observed during the second shot. Note that the value of 1630 °C is the corrected measurement upper limit of our IR sensor system. Consequently, there exist temperature saturation peaks in the transient temperature profiles when the measured temperature exceeds the upper limit. Interestingly, ultrafast reduction within 1 ms was observed at a higher energy density of ≈11.0 J cm^−2^, with no shoulder region detected (Figure S4a, Supporting Information). Based on the optical reduction behavior of GO, similar experiments were conducted for BA@GO to further investigate B doping mechanisms. BA@GO was irradiated with a controlled number of shots (from one to four) at an energy density of 4.9 J cm^−2^ (Figure S4b, Supporting Information). Similarly to the case of GO, the maximum temperature increased with increasing pulse number, which was indicative of gradual reduction progress, as supported by ex situ XPS results (Figure S4c–e, Supporting Information). Notably, BA@GO required more light energy than GO to reach the analogous temperature range (suggested in Figure 1b), which was attributed to the presence of a BA particle overlayer on GO in the former case. BA particles on the GO surface partially blocked its direct light absorption, thus attenuating photothermal effects. Interestingly, the temperature obtained after the first shot stayed rather low (380 °C). Given that BA has a boiling point of 300 °C, the corresponding shoulder part was mainly ascribed to the partial vaporization of BA on GO sheets to increase their light absorption efficiency. Despite the temperature rise to 847.3 °C after the fourth shot, no significant doping was achieved (Figure S4f–h, Supporting Information), that is, the activation temperature for B doping into GO sheets was much higher than this value. To further probe the mechanism of photothermal effect–induced reduction and doping, higher‐energy pulses (≈11.0 J cm^−2^) were employed (Figure 1d). As expected, during the first shot, the temperature rapidly increased within 1 ms and stayed at ≈400 °C for 5 ms, which suggested BA vaporization. The absence of a shoulder peak after the first shot indicated that BA crystallites covering the GO surface were sufficiently vaporized for the effective absorption of light by GO. As reduction was not complete, the second‐shot peak could not reach the corrected measurable limit (1630 °C). However, BA vaporization and partial reduction resulted in increased photothermal effect–based heat generation, which allowed for further reduction at temperatures above 1630 °C. As shown in Figure S4a, Supporting Information, higher light energy densities resulted in a shorter process time within a single shot. Therefore, numerous shots with sufficiently high energy densities (>12.0 J cm^−2^) were applied to BA@GO to estimate the activation temperature for millisecond‐scale B doping (Figure 1e). The slow temperature increase in the range of 250–450 °C and the subsequent faster rise were ascribed to GO reduction and BA vaporization, respectively. The observation of GO reduction and BA vaporization as separate processes was attributed to the low degree of BA reduction on GO sheets. Maximum temperatures of 944.0, 1185.5, 1444.8, and 1605.3 °C were reached for samples irradiated with a single shot at energy densities of 14.1, 21.9, 25.8, and 31.3 J cm^−2^, respectively. Changes in the valence states of B and C were probed by ex situ XPS (Figure S5, Supporting Information). The intensity of BCO_2_ peaks in B 1s spectra increased as the temperature went up to 1400 °C, whereas no significant change was observed below 1400 °C (Figure S5a–d, Supporting Information). Furthermore, in addition to BC_2_O and BC_3_ peaks, C—O and C=O signals due to further reduction and decomposition were detected (Figure S5e–h, Supporting Information). Therefore, the activation temperature for B doping was assumed to exceed 1400 °C. To further explore doping mechanisms, much higher temperatures of >1630 °C, that is, exceeding the measurable range of the IR sensor, were generated on BA@GO (Figure S6, Supporting Information). Figure S6d–g, Supporting Information, shows the corresponding B 1s spectra, revealing that with increasing temperature, BCO_2_ peak intensity dramatically decreased, and BC_4_‐related peaks emerged. As a result, we concluded that temperatures above 1600 °C can induce BCO_2_ unit cleavage and BC_4_ moiety formation. Based on the results of ex situ XPS analysis, we suggested the millisecond bond engineering of temperature‐dependent sequential mechanisms in ambient air based on photothermal effects (Figure 1f and Figure S7, Supporting Information). In addition, the resultant ex situ XPS data indicates that the photothermal treatment can control the oxygen content and functionality of the samples by modulation of conditions (Figure S8, Supporting Information).

To demonstrate the feasibility of the adopted approach for large‐area synthesis, an IPL lamp with a large beam size (height = 75 mm, width = 150 mm) was employed (Figures S9 and S10, Supporting Information). A high beam uniformity of >97% was obtained through the use of a unique parabolic reflector system. High beam uniformity and large beam area are well suited for use in automated conveyor belts systems, which are considered to be suitable for roll‐to‐roll mass production of flexible electronics.^[^[qv: 19,20,35]^]^ Glass substrates coated with BA@GO were placed on the IPL equipment stage at a distance of 5 mm from the quartz plate of the Xe lamp (Figure S9a,b, Supporting Information). As the above distance was shorter than that (4 cm) used for the in‐depth study, doping and reduction were realized at a much lower energy density in the former case. All IPL treatments were performed at an energy density of 1.1 J cm^−2^, a constant voltage of 300 V, and a duration time of 3 ms. After a single shot, the dark brown film turned black (Figure S11, Supporting Information), which was indicative of successful large‐area synthesis of B@rGO. Residual boron oxide in B@rGO films was removed by 30‐min dipping of substrates into deionized water at 60 °C (Figure S12, Supporting Information).

**Figure 2:**
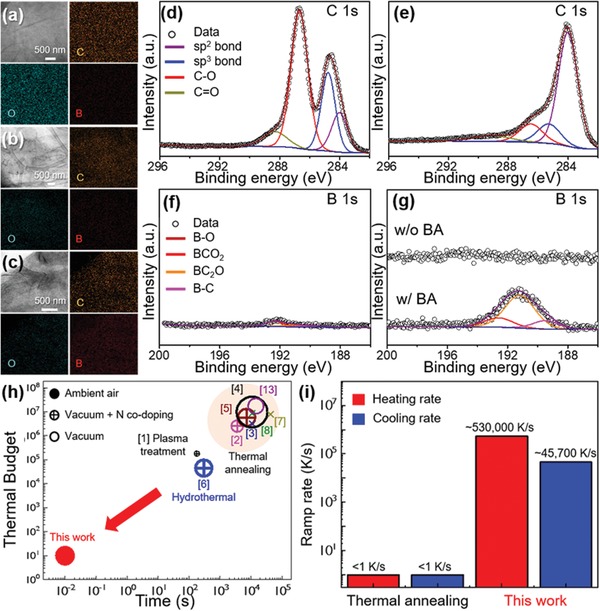
TEM images and EDS overlay mappings of a) pristine GO, b) rGO, and c) B@rGO. High‐resolution C 1s spectra of d) pristine GO, and e) B@rGO and f) B 1s spectrum of pristine GO. g) High‐resolution B 1s spectra of rGO (upper, without BA) and B@rGO (lower, with BA). h) Thermal budget–process time curves describing the B doping of rGO for various doping approaches, with circle size indicating dopant concentration. The corresponding references are listed in Table S1, Supporting Information. i) Heating and cooling rates of thermal annealing and low‐thermal‐budget approaches.

**Figure** **2**a–c shows transmission electron microscopy (TEM) images and energy‐dispersive X‐ray spectroscopy (EDS) overlay mappings of pristine GO, rGO, and B@rGO, respectively. EDS revealed that the initially high content of O (sky blue) in GO decreased in rGO and B@rGO (i.e., after IPL irradiation), in line with the successful reduction of GO, and also showed that B was homogeneously distributed on the B@rGO surface (Figure 2c). To further identify the presence and valence states of B and understand changes due to simultaneous B doping and reduction, GO and B@rGO were subjected to XPS analysis. Figure 2d,e shows high‐resolution C 1s spectra of GO and B@rGO, respectively, revealing the presence of characteristic C sp^2^ (284.0 eV) and C sp^3^ (284.8 eV) peaks.^[^[qv: 36]^]^ However, the intensities of C—O and C=O peaks (red lines) dramatically decreased upon irradiation, which indicated GO reduction to rGO. To clearly define B doping, high‐resolution B 1s spectra of GO, rGO, and B@rGO were acquired (Figure 2f,g, respectively). After IPL treatment of BA@GO, the intensities of peaks related to B–C bonds dramatically increased, that is, B doping was successfully realized within 3 ms in ambient air. As expected, rGO obtained from GO in the absence of BA contained no B. The presence of B in B@rGO was also confirmed by the shift of X‐ray diffraction (XRD) peaks to higher angles and the change of the (002)‐plane *d*‐spacing from 3.54 to 3.42 Å, as reported previously (Figure S13, Supporting Information).^[^[qv: 37]^]^ The high resolution B 1s spectra in Figure 2g featured BCO_2_, BC_2_O, and BC_3_ peaks at 192.58, 191.19, and 189.46 eV, respectively.^[^[qv: 38]^]^ Notably, B–C bonds in the carbon structure mainly corresponded to BC_2_O units. Similar phenomena have been observed for the synthesis of B‐doped GO by long‐term (>3 h) high‐temperature (>800 °C) annealing in a reducing atmosphere.^[^[qv: 39,40]^]^ The concentration of B doped into rGO was estimated as 3.56 at% (Figures S14 and S15, Supporting Information). Even though IPL‐assisted B doping featured a short on‐state time (3 ms) in ambient air, the dopant concentration was comparable to or even higher than that in B@rGO synthesized by conventional (time‐ and energy‐consuming) thermal processes (Table S1, Supporting Information). Notably, our approach allowed doping and reduction to be accomplished without substrate damage even in cases of flexible polymer substrates (Figure S16, Supporting Information). To confirm the effects of IPL irradiation energy on doping, another sample was irradiated at 5.6 J cm^−2^, and energy density was shown not to influence dopant concentration (Figure S17, Supporting Information). Notably, IPL treatment resulted in the formation of BC_4_ units, which could not be previously achieved by conventional thermal methods (Table S1, Supporting Information). Figure 2h shows the calculated thermal budget and process time of various doping approaches, with circle sizes representing dopant concentration. The thermal budget was obtained by calculating the area of the temperature‐time curve for a certain process or process sequence and was considered to be the total amount of thermal energy consumed to perform doping. Normally, a lower thermal budget allows for cost‐effective and productive thermal processes, leading to energy‐efficient manufacturing. As depicted above, thermal annealing has a large thermal budget due to inefficient heat conduction in furnace systems. Besides, all processes in the thermal budget‐process time curve are only available in vacuum systems, restricting the facile and universal expansion of the strategy to other types of heteroatom doping. Remarkably, photothermal doping featured a 10^6^‐fold lower thermal budget (even in ambient air) than other methods requiring vacuum or reducing‐atmosphere processing, thus allowing for strikingly facile and ultrafast doping. This exceptional performance was attributed to the extremely high heating (5.30 × 10^5^ °C s^−1^) and cooling (4.57 × 10^4^ °C s^−1^) rates of the photothermal method compared to those of conventional thermal annealing techniques (Figure 2i).

**Figure 3:**
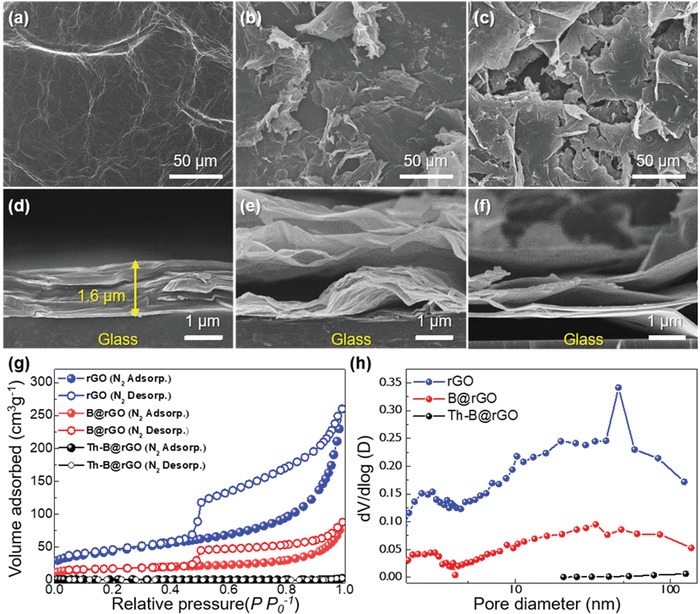
SEM images of a) pristine GO, b) rGO, and c) B@rGO. Cross‐sectional SEM images of d) pristine GO, e) rGO, and f) B@rGO. g) N_2_ adsorption–desorption isotherms and h) pore size distributions of rGO, B@rGO, and Th‐B@rGO.

Surface area is an important parameter determining electrochemical properties, photocatalytic activity, and reactivity toward gas molecules.^[^[qv: 28,29]^]^ The surface morphologies of pristine GO, rGO, and B@rGO were observed by scanning electron microscopy (SEM) (**Figure** **3**a–c) and were shown to be typical of GO, corresponding to planar surfaces with wrinkled and folded features. After single‐shot IPL irradiation (300 V, 3 ms, 1.1 J cm^−2^), open porosity development and a surface area increase were observed for rGO and B@rGO, which facilitated diffusion and reactivity. Interestingly, the porous framework of rGO and B@rGO did not appear in thermally treated B@rGO. Cross‐sectional SEM imaging (Figure 3d–f) indicated the occurrence of exfoliation and floating for rGO and B@rGO, which increased the exposure of graphene sheets with a high surface area. Photothermal effects could be not manifested down to graphene sheets in the vicinity of glass substrates, as all light energy had been consumed. However, upon photothermal treatment, graphene sheets near glass substrates swelled to produce an open pore structure. Figure S18, Supporting Information, further shows that this exfoliation and floating of graphene sheets was accomplished in all parts. The structural features of GO, rGO, and B@rGO were further probed by Raman spectroscopy (Figure S19, Supporting Information).

Porosities and Brunauer–Emmett–Teller (BET) surface areas of rGO, B@rGO, and thermally reduced and doped rGO (Th‐B@rGO) were determined from N_2_ adsorption–desorption isotherms recorded at 77 K (Figure 3g). Specifically, the BET surface areas of rGO and B@rGO were determined as 153.86 and 60.59 m^2^ g^−1^, respectively. As B@rGO was obtained by irradiation of BA@GO, a fraction of light energy was consumed for B doping, and the corresponding increase in surface area was less than that of pristine rGO. The low surface area of Th‐B@rGO (1.76 m^2^ g^−1^) was attributed to the noticeable difference in temperature ramping rates between IPL and thermal treatment methods. In contrast to the case of thermal treatment, the abrupt temperature rise observed for IPL treatment resulted in a rapid increase of pressure inside graphene sheets due to the decomposition of GO oxygenated groups, leading to graphene sheet exfoliation and floating (Figure S18, Supporting Information).^[^[qv: 41]^]^ In turn, ultrafast reduction and doping due to IPL irradiation could be an effective strategy of surface area increase. The isotherms of rGO and B@rGO were classified as type‐IV (hysteresis loop at *P*/*P*_0_ ranged from 0.5 to 1.0), indicating the presence of abundant pores (mainly mesopores, 2–50 nm), as verified by Barrett–Joyner–Halenda pore size distributions (Figure 3h). On the contrary, Th‐B@rGO exhibited a negligible mesopore distribution.

The aforementioned findings clearly confirm the success of photothermal LTB doping and reduction of GO, showing that the resulting B@rGO with B‐doped networks of sp^2^ carbons and a high surface area can be used in surface reaction–dominated applications such as gas sensing layers, supercapacitor electrodes, and photocatalysts.^[^[qv: 6,39,42,43]^]^ Herein, pristine rGO and B@rGO were used as room‐temperature gas sensing layers. The sensors were exposed to 1–20 ppm NO_2_ in dry conditions, with the corresponding normalized resistance changes (∆*R*/*R*_air_) shown in Figure S20a,b, Supporting Information. Compared with pristine rGO, B@rGO showed a 3.1‐fold enhanced response to 10 ppm NO_2_, which was ascribed to the presence of B‐doped active reactions sites in the latter case, as reported previously.^[^[qv: 13,42]^]^ In addition, B@rGO exhibited a more reliable sensing behavior with better reversibility upon exposure to 10 ppm NO_2_ for 15 cycles, whereas pristine rGO showed a continuous downward drift of baseline resistance (Figure S20c, Supporting Information). B@rGO sensors showed excellent selectivity to NO_2_ in the presence of interfering molecules such as hydrogen, toluene, ethanol, hydrogen sulfide, ammonia, and carbon monoxide (Figure S20d, Supporting Information).

**Figure 4:**
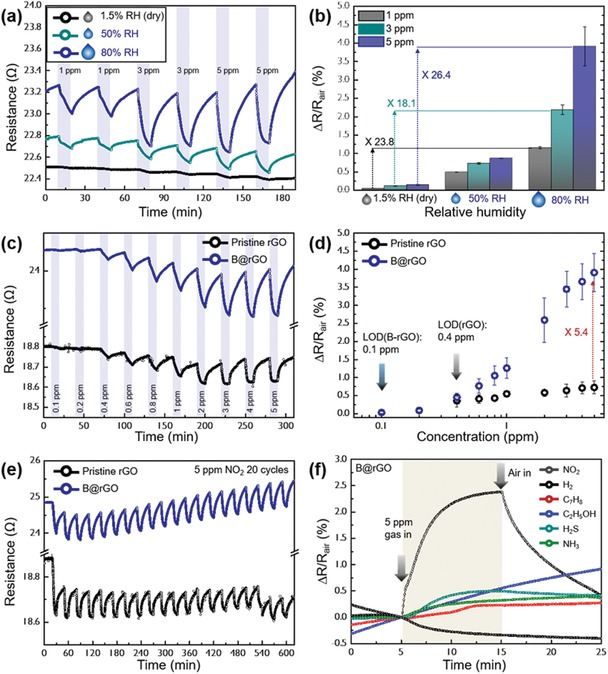
a) Dynamic resistance transients and b) sensitivities of B@rGO to 1, 3, and 5 ppm NO_2_ at 1.5, 50, and 80% RH. c) Dynamic resistance transients of pristine rGO and B@rGO and d) sensitivities of the above materials to 0.1–5 ppm NO_2_ at 80% RH. e) Reliability of pristine rGO and B@rGO sensors determined by repetitive exposure to 5 ppm NO_2_ at 80% RH for 20 cycles. f) Responses of B@rGO to six different gases (5 ppm NO_2_ at 80% RH).

As GO‐based byproducts possess residual oxygenated groups sensitive to humidity, NO_2_ sensing performance was further tested for three different relative humidity (RH) conditions, that is, RH = 1.5 (dry), 50, and 80%, and the corresponding normalized resistance changes were calculated (**Figure** **4**a,b). Compared to the case of 1.5% RH, B@rGO sensors show a considerable improvement of responses to 1 and 5 ppm NO_2_ (23.8‐ and 26.4‐fold) at 80% RH. For pristine rGO, the corresponding enhancement factors equaled 17.2 and 3.6, respectively (Figure S21a,b, Supporting Information). We further compared the sensing performances of pristine rGO and B@rGO toward 0.1–5 ppm NO_2_ at 80% RH and calculated the corresponding normalized resistance changes (Figure 4c,d), demonstrating that stable response and recovery (i.e., good reversibility) were observed for both B@rGO and pristine rGO. Compared to that of pristine rGO, the response of B@rGO toward 5 ppm NO_2_ was enhanced 5.4‐fold because of the abundant B active reaction sites of the latter material. The NO_2_ detection limits of B@rGO and pristine rGO were determined as 100 and 400 ppb, respectively, and the above response enhancement well matched the results obtained for 1.5% RH. Pristine rGO and B@rGO showed notable reliability during exposure to 5 ppm NO_2_ at 80% RH over 20 cycles (Figure 4e). Moreover, the B@rGO sensor exhibited excellent NO_2_ selectivity against hydrogen, toluene, ethanol, hydrogen sulfide, and ammonia as interferents (Figure 4f). The long‐term performance of B@rGO was monitored by comparing the response of a fresh sensor with that of a two‐month‐used sensor to 1–5 ppm NO_2_. The deviation between these responses was less than 23%, indicating good long‐term stability (Figure S22, Supporting Information). To emphasize the superiority of the IPL irradiation method, we further compared the NO_2_ sensing properties of non‐IPL‐treated pristine GO with those of Th‐B@rGO obtained by thermal annealing (Figure S23a,b, Supporting Information). Both sensors featured negligible or poor responses toward 0.1–5 ppm NO_2_ at 80% RH. Note that even if residual B_2_O_3_ particles were present on GO sheets, they would not be reactive toward NO_2_ gas by themselves (Figure S24, Supporting Information). In this regard, B@rGO obtained by IPL irradiation demonstrated good NO_2_ sensing characteristics that further improved with increasing RH. The above enhancement was ascribed to the synergistic effects of GO reduction/B‐doping via IPL irradiation as well as to the humidity‐assisted improvement of NO_2_ sensing characteristics, which has not been deeply considered previously. Both GO and rGO possess abundant hydroxyl and carboxyl surface functional groups, and have therefore been used for effective humidity sensing based on the easy formation of hydrogen bonds between the above functional groups and water molecules.^[^[qv: 44,45]^]^ Under highly humid conditions, water molecules are readily adsorbed on the B@rGO surface, facilitating the ionization of surface carboxyl groups to form COO^–^ and H^+^.^[^[qv: 46]^]^ The COO^–^ groups act as hole traps, generally leading to hole depletion in *p*‐type carbon materials, that is, to a baseline resistance increase. Another possible mechanism is the band gap increase due to the adsorption of water molecules on the grain boundary or defective sites of graphene flakes, which increases resistance.^[^[qv: 47,48]^]^ The humidity sensing properties of GO, rGO, and B@rGO are presented in Figure S25, Supporting Information. As expected, GO with abundant oxygenated groups was more sensitive to humidity (6.1–99.9% RH) than rGO and B@rGO. The higher humidity sensitivity of B@rGO compared to that of rGO was attributed to the higher amount of residual oxygenated functional groups in the former case after an identical IPL treatment (Figure S17a,b, Supporting Information). In this regard, B@rGO exhibited a larger enhancement of humidity‐dependent NO_2_ sensing performance than pristine rGO (Figure 4; Figure S21, Supporting Information).

**Figure 5:**
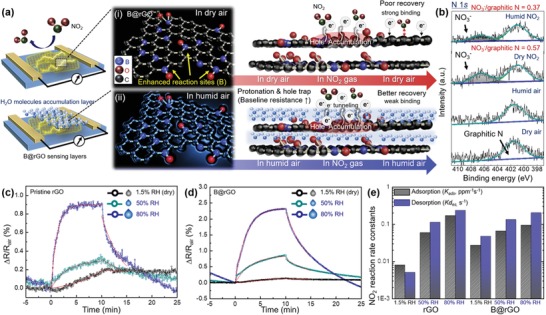
a) Illustration of the NO_2_ sensing mechanism of B@rGO in i) dry air and ii) humid air. b) Ex situ N 1s XPS spectra of B@rGO recorded in dry and humid air before and after exposure to NO_2_. c) Response and recovery fitting curves and raw response and recovery curves for NO_2_ sensing by c) pristine rGO and d) B@rGO. e) Rate constants for the NO_2_ reaction determined at 1.5%, 50%, and 80% RH.

The mechanism of NO_2_ sensing by B@rGO can be explained by considering the effects of B doping and humidity, as illustrated in **Figure** **5**a. Compared to pristine rGO, B@rGO possesses B‐doped active sites in the forms of BC_3_, BCO_2_, and BC_2_O, as confirmed by XPS analysis (Figure 2g). Such sites in the carbon matrix are known to provide stronger binding and more abundant sites for reactions with NO_2_ molecules, which leads to higher modulation of resistance and a higher response.^[^[qv: 13]^]^ In dry air, NO_2_ molecules are likely to be strongly adsorbed on either hydroxyl groups or B‐doped active sites of B@rGO, attracting electrons from the B@rGO surface.^[^[qv: 49]^]^ In addition, NO_2_ molecules can react with physisorbed O_2_ (2NO_2_(gas) + O_2_(gas) + 2*e* ^−^ → 2NO_3_^−^(ads)) to form hole‐accumulation regions and dramatically decrease resistance (Figure 5a–i).^[^[qv: 50]^]^ NO_3_^−^ ions tend to be adsorbed and stacked on the surface of sensing layers, and hence, imperfect recovery behavior is observed, as in cases of other conventional room‐temperature carbon‐based sensors.^[^[qv: 51–53]^]^ Generally, recovery kinetics can be improved using cleaning techniques such as UV light irradiation to remove adsorbed gas analytes.^[^[qv: 54,55]^]^ In our case, simple humidity control could dramatically enhance reversibility during NO_2_ sensing. In the employed system, water molecules were thought to i) promote hole trap formation to increase baseline resistance and thus contribute to the higher resistance variation during NO_2_ injection and ii) partially hinder the direct chemisorption of NO_2_ on B@rGO sensing layers to promote NO_2_ reaction reversibility. The humidity‐assisted enhancement of the NO_2_ sensing properties of heteroatom‐doped rGO has been considered for the first time, especially at room temperature. As electrons can readily tunnel through the H_2_O layer, hole accumulation regions on B@rGO sensing layers can be effectively tuned during NO_2_ injection and elimination, similarly to the case of 1.5% RH.^[^[qv: 56]^]^ The enhanced response to NO_2_ at high RH was partially attributed to the humidity‐dependent interlayer (*d*‐) spacing increase, which favored the penetration of gases for active surface reactions, as reported previously.^[^[qv: 57]^]^ Additionally, UPS analysis was conducted to identify the work functions of GO (5.55 eV), rGO (4.45 eV), and B@rGO (4.60 eV), as shown in Figure S26, Supporting Information. The elimination of the oxygenated functional groups in GO, during the IPL irradiation, releases the electrons back to rGO, inducing a reduction of the work function. Additionally, the energy‐state shift (0.15 eV) of the work function, between rGO and B@rGO, indicates a p‐type doping effect, which is attributed to the enhanced NO_2_‐sensing performance.

The effects of humidity on NO_2_ adsorption and desorption kinetics were probed by ex situ XPS analysis of B@rGO sensing layers in dry air, humid air, dry NO_2_, and humid NO_2_ (Figure 5b). All samples exhibited a graphitic N peak at ≈401 eV.^[^[qv: 58]^]^ Typically, NO_2_ molecules adsorb on rGO in the form of NO_3_^–^ and remain on the surface if recovery is not fully conducted, as mentioned previously. After exposure to dry NO_2_ gas, the NO_3_^–^ to graphitic N peak intensity ratio was determined as 0.57, decreasing to 0.37 upon exposure to NO_2_ at 80% RH, which corresponded to a considerable improvement in NO_2_ desorption kinetics. Adsorption (*k*_ads_) and desorption (*k*_des_) rate constants were calculated by fitting the response versus time curves of rGO and B@rGO at 1.5%, 50%, and 80% RH as follows:

(2) $$R\left(t\right)\;\;\text{for}\;\;{\text{NO}}_{2}\;\;\text{adsorption}\;\;=\;\;R_{\text{max}}\cdot \frac{C_{a}K}{1\;\;+\;\;C_{a}K}\left(1\;\;-\;\;\exp\left[-\frac{1\;\;+\;\;C_{g}K}{K}\cdot k_{\text{ads}}t\right]\right)$$

(3) $$R\left(t\right)\;\text{for}\;{\text{NO}}_{2}\;\text{desorption}=R_{\text{air}}\;\exp\left[-k_{\text{des}}t\right]$$

where *R*_air_ is the response in baseline air, *R*_max_ is the maximum sensor response, and *C_a_* is the concentration of NO_2_. Note that response and recovery kinetics were calculated based on the mass action law of NO_2_ adsorption reactions under the assumption that the response is proportional to the adsorbed gas amount.^[^[qv: 59]^]^ As a result, a noticeable enhancement in reaction kinetics was observed with increasing humidity for both rGO and B@rGO sensing layers. Specifically, the adsorption and desorption rate constants of B@rGO obtained at 80% RH exceeded those obtained at 1.5% RH 3.5‐ and 4.3‐fold, respectively (Figure 5c–e). These results demonstrate the synergistic effects of i) the simultaneous light‐induced reduction and B doping of GO and ii) controlled humidity, contributing to the response and reversibility improvement of room‐temperature B@rGO sensing layers.

## 3 Conclusion

Herein, we successfully demonstrated a low‐thermal‐budget mass‐production‐suitable synthesis of heteroatom‐doped rGO through intense pulse light irradiation in ambient air within <10 ms and probed photothermally induced B‐doping processes by millisecond‐scale transient temperature profiling and ex situ XPS. GO reduction was followed by the formation of B–C bonds, and a substantial extent of B‐doping (≈3.60 at% B) was achieved. B@rGO with abundant reaction sites was used as an effective room‐temperature NO_2_ gas sensing layer, featuring significantly enhanced response and reaction kinetics compared to those of pristine GO and rGO, especially under controlled humidity conditions. Thus, intense pulsed light–assisted optical engineering was concluded to be a facile and general strategy for carbon matrix doping with heteroatoms. As an example, B@rGO with abundant B active sites was demonstrated to act as a high‐performance chemiresistor with effectively tunable physicochemical properties.

## 4 Experimental Section

##### BA@GO Coating on Substrates

Boric acid (H_3_BO_3_. >99%) and GO dispersed DI solution (5 mg mL^−1^) were purchased from Sigma‐Aldrich (St. Louis, USA). All chemicals were used without further purification. For boron doping into GO, 20 mg of boric acid was mixed with 2 mL of GO dispersed DI solution and ultra‐sonicated for an hour to obtain GO solution with boric acid (BA@GO) uniformly distributed. Then, the mixture of 10 µL was drop‐coated on the glass substrate with micropipette several times and dried in ambient air for an hour.

##### Optical Doping and Reduction by IPL Irradiation

A Xenon flash lamp (PLT, Photocura) was employed to form reduced GO sheets (rGO) and B doped reduced GO sheets (B@rGO) on glass substrates. The spectrum of a light source ranged from about 300 to 1000 nm (Figure S10a, Supporting Information), especially exhibiting high intensity from 400 to 700 nm. The light energy can be adjusted by tuning the applied voltage, pulse on/off time, pulse number and sample distance from the quartz. The glass substrates coated with GO and BA@GO were placed with 5 mm distance from the quartz (Figure S9b, Supporting Information). The pulse on‐time was set as 3 ms with a constant applied voltage of 300 V to the lamp to maintain the flash light energy as 1.1 J cm^−2^. A single light pulse generated from a Xenon lamp was irradiated onto the GO and BA@GO sheets for the in situ synthesis of rGO and B@rGO. To further verify effects of light irradiation on polymer substrates, colorless polyimide (cPI) films were prepared. Following the same coating method, BA@GO‐coated cPI film was prepared and treated with the identical IPL irradiation condition. Interestingly, no damage on the polymer substrate was observed, suggesting the feasibility of in situ synthesis of B@rGO on the flexible substrate for the flexible platform (Figure S16a,b, Supporting Information).

##### Preparation of B_2_O_3_

To obtain B_2_O_3_ particles, boric acid was placed in an alumina crucible and sintered through two step heating methods in a box furnace. First, boric acid was annealed at 130 °C for 30 min with ramping rate of 2 °C min^−1^. Afterwards, the subsequent annealing was performed at 330 °C for 1 h with ramping rate of 2 °C min^−1^ from 130 °C to obtain high purity B_2_O_3_.

##### Characterization

The morphologies and cross‐section images of GO, rGO, and B@rGO were characterized with scanning electron microscopy (SEM, XL‐30 SFEG, Philips) and field emission transmission electron microscopy (FETEM, Tecnai G2 F30 S‐Twin, FEI). The crystal structures of the samples were investigated using X‐ray diffraction (XRD, D/MAX‐RC 12 kW, Rigaku) with Cu K*_a_* (*l* = 1.54 Å) radiation. The chemical elements and bonding states of GO and B@rGO were evaluated by X‐ray photoelectron spectroscopy (XPS, Sigma Probe, Thermo VG Scientific) with Al K*_a_* radiation (1486.6 eV). Raman spectra of rGO, B@rGO, and Th‐B@rGO were analyzed by with 532 nm laser. Measurement of the surface areas (rGO, B@rGO, and Th‐B@rGO) were performed using the Brunauer–Emmett–Teller theory (BET, ASAP2020, Micromeritics).

##### IR Sensor System

IR information with wavelength of 2.3 µm from samples is measured by a IR thermometer (CTlaser 3MH2, Optris). The IR sensor can detect from 200 °C up to 1500 °C. It should be noted that the base temperature is displayed as 200 °C due to the detectable temperature range and emissivity is set at unity when temperature measurement is performed. After emissivity correction, the measurement upper limit of the sensor system increases up to about 1630 °C, which is the temperature at which the temperature saturation peaks in the transient temperature profile are observed. For accurate measurements, samples should be located 15 cm distant from the IR sensor (See Figure S3a, Supporting Information). Measured analogue signals are corrected and converted to digital signals by a data acquisition board (NI USB‐6341 X series Multifunction DAQ, National Instruments). Finally, measured data are shown in a form of temperature (For more detail, see Figure S3, Supporting Information).

##### Gas Sensing Characterization

At first, the sensing materials, that is, GO, rGO, and B@rGO were coated on the Al_2_O_3_ substrate, patterned with two parallel electrodes having width and distance of 25 and 70 µm, respectively (Figure S27a, Supporting Information). Note that rGO and B@rGO samples were obtained by a single flash irradiation with large‐area beam. The duration time of the flash shot was set as 10 ms with a constant applied voltage of 300 V to the lamp to maintain the flash light energy as 3.3 J cm^−2^. The sensing properties were evaluated by using homemade testing equipment (Figure S27b, Supporting Information). By using a data acquisition system (34972A, Agilent) with a 16‐channel multiplexer (34902A, Agilent), the resistance of the sensors were measured every 4 s and the values were converted into the normalized resistance change value, that is, ∆*R*/*R*_air_, where ∆*R* denotes *R*_air_ − *R*_gas_, *R*_gas_ is the baseline resistance in gas, and *R*_0_ is the baseline resistance in air. The relative humidity (RH) level was controlled by using a humidity generator. All the sensors were stabilized in the baseline air for 2 h prior to the sensor tests. The NO_2_ concentration was controlled within 0.1–20 ppm with a cyclic exposure of 10 min NO_2_ followed by 20 min baseline air, respectively. All the sensing measurements were conducted at room temperature.

## Conflict of Interest

The authors declare no conflict of interest.

## Supporting information

Supporting Information
