# Supplementary material for: Low‐Thermal‐Budget Doping of 2D Materials in Ambient Air Exemplified by Synthesis of Boron‐Doped Reduced Graphene Oxide
Source: Adv Sci (Weinh). 2020 Feb 22;7(7):1903318. doi: 10.1002/advs.201903318 (PMC7140995; doi:10.1002/advs.201903318)
Supplement: Supplementary file 1 — Supporting Information [file ADVS-7-1903318-s001.pdf]

Copyright WILEY-VCH Verlag GmbH & Co. KGaA, 69469 Weinheim, Germany, 2020.

Supporting Information

for *Adv. Sci.,* DOI: 10.1002/advs.201903318

Low-Thermal-Budget Doping of 2D Materials in Ambient Air Exemplified by Synthesis of Boron-Doped Reduced Graphene Oxide

Jun-Hwe Cha, Dong-Ha Kim, Cheolmin Park, Seon-Jin Choi, Ji-Soo Jang, Sang Yoon Yang, Il-Doo Kim, and Sung-Yool Choi**

Copyright WILEY-VCH Verlag GmbH & Co. KGaA, 69469 Weinheim, Germany, 2020.

Supporting Information

Low-Thermal-Budget Doping of 2D Materials in Ambient Air Exemplified by Synthesis of Boron-Doped Reduced Graphene Oxide

Jun-Hwe Cha,† Dong-Ha Kim,† Cheolmin Park, Seon-Jin Choi, Ji-Soo Jang, Sang Yoon Yang, Il-Doo Kim, and Sung-Yool Choi**

Jun-Hwe Cha, Dr. Cheolmin Park, Dr. Sang Yoon Yang, Prof. Sung-Yool Choi
School of Electrical Engineering,
Graphene/2D Materials Research Center,
Center for Advanced Materials Discovery towards 3D Displays,
Korea Advanced Institute of Science and Technology (KAIST), 291 Daehak-ro, Yuseong-gu, Daejeon 34141, Republic of Korea
*Corresponding author. Email: sungyool.choi@kaist.ac.kr (S.-Y. Choi)

Dong-Ha Kim, Ji-Soo Jang, Prof. Il-Doo Kim
Department of Materials Science and Engineering,
Korea Advanced Institute of Science and Technology (KAIST), 291 Daehak-ro, Yuseong-gu, Daejeon 34141, Republic of Korea
*Corresponding author. E-mail: idkim@kaist.ac.kr (I.-D. Kim)

Prof. Seon-Jin Choi
Department of Materials Science and Engineering,
Hanyang University, Wangsimni-ro, Seongdong-gu, Seoul 04763, Republic of Korea

†These authors contributed equally to this work.

Table of Contents

S1. XPS analysis of GO and BA@GO in the vicinity of C 1s and B 1s peaks.
S2. Optical microscopy and SEM images of BA@GO
S3. Optical image of IR sensor system and temperature-time curve during IPL exposure in air.
S4. Temperature-time curve and ex-situ XPS analysis of GO and BA@GO.
S5. IPL power dependent ex-situ XPS analysis of BA@GO in the vicinity of B 1s and C 1s.
S6. Temperature-time curve and ex-situ XPS analysis of BA@GO with differnt IPL power.
S7. Ex-situ XPS analysis of B@rGO with different IPL conditions and suggested reaction mechanisms.
S8. Ex-situ XPS analysis of GO, rGO, and B@rGO in the vicinity of C 1s.
S9. Camera images of the IPL equipment.
S10. Spectral information of xenon flash lamp.
S11. Camera images of GO and rGO before and after IPL treatment.
S12. XPS spectra of B@rGO in the vicinity of B 1s peaks before and after DI cleaning.
S13. XRD analysis of rGO and B@rGO.
S14. XPS survey spectra of B@rGO.
S15. XPS spectra of B@rGO in the vicinity of C 1s, O 1s, B 1s, and N 1s peak.
S16. Camera images of B@rGO synthesized on flexible polymer substrates.
S17. XPS analysis of rGO and B@rGO synthesized by large-area beam.
S18. Cross-section SEM images of rGO and B@rGO.
S19. Raman spectra of GO, rGO and B@rGO.
S20. Sensing properties of rGO and B@rGO in dry condition.
S21. NO_2 sensing properties of rGO in different humidity
S22. Long-term stability of B@rGO upon exposure to 1–5 ppm NO_2
S23. NO_2 sensing properties of GO and Th-B@rGO.
S24. NO_2 sensing properties of B_2O_3.
S25. Humidity sensing properties of GO, rGO, and B@rGO.
S26. UPS analysis of GO, rGO, and B@rGO.
S27. Schematic illustration of the alumina sensor substrate and the gas sensor measurement system.
Table S1. State-of-the-art publications on B doped rGO .

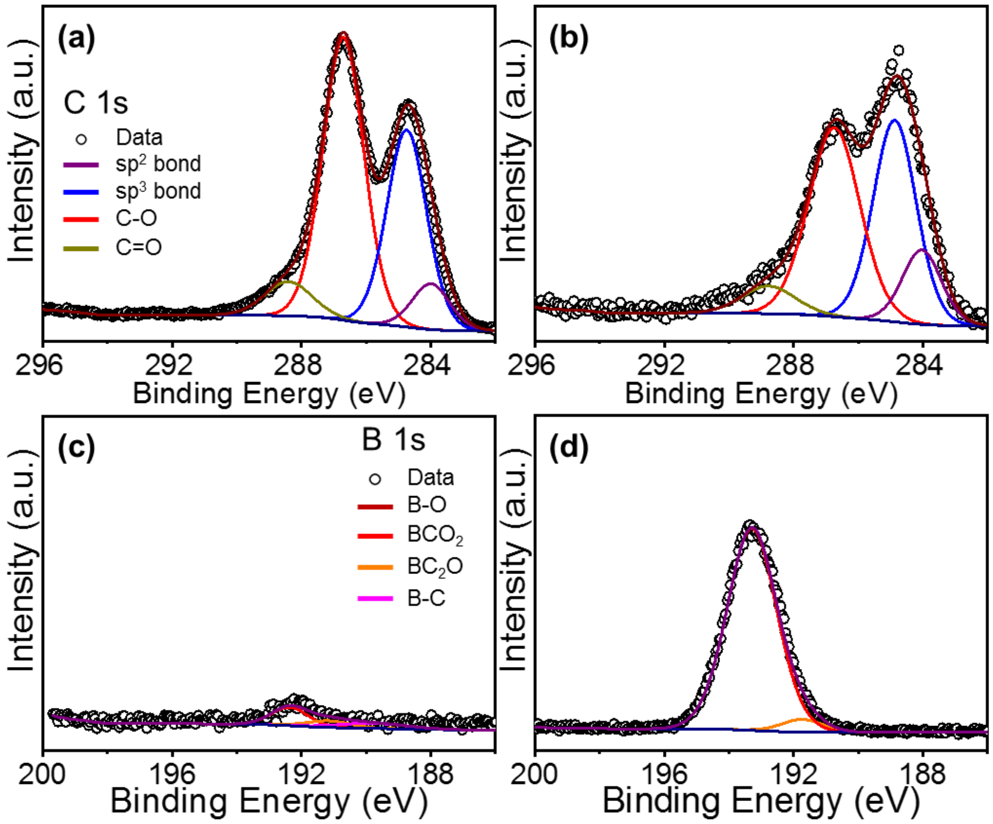

Figure S1. X-ray photoelectron spectroscopy (XPS) spectra of pristine GO and BA@GO: High-resolution spectra in the vicinity of the C 1s for (a) pristine GO, and (b) BA@GO, B 1s for (c) pristine GO, (d) BA@GO.

Figure S1a shows that pristine GO sheets possess abundant oxygen functional groups comprising of C-O and C=O bonds. In addition, it was found that neglilable boron bonds exist from the high-resolution spectra in the vicinitiy of the B 1s (Figure S1c). For doping source, boric acid was introduced by mixing with pristine GO dispersion in deionized (DI) water. After the mixing, partial reduction occurred mainly with the removal of C-O bond. Interestingly, XPS analysis strongly confirmed that decomposition of oxygen functional groups and BCO_2 bonds formation occur at the same time.

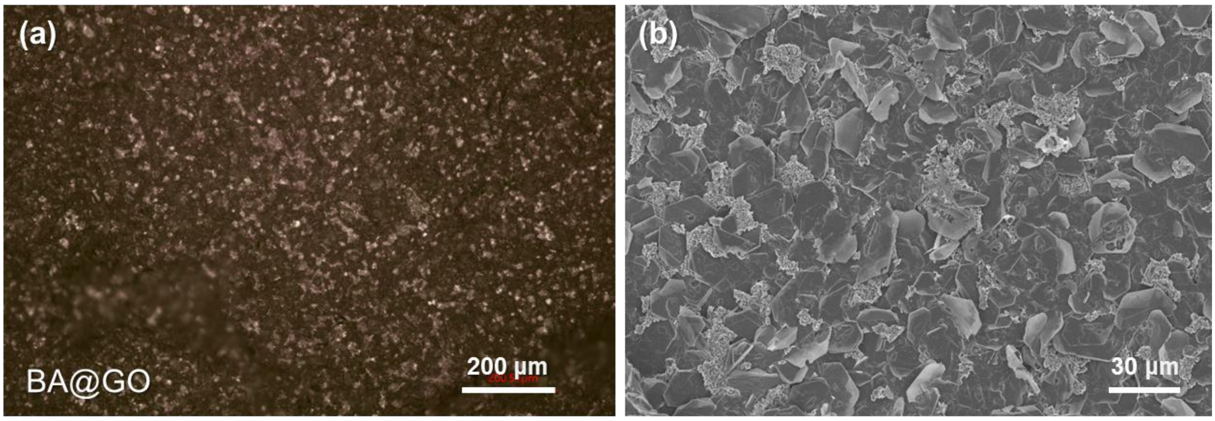

Figure S2. (a) Optical microscopy image of BA@GO, (b) Scanning electron microscopy (SEM) images of BA@GO.

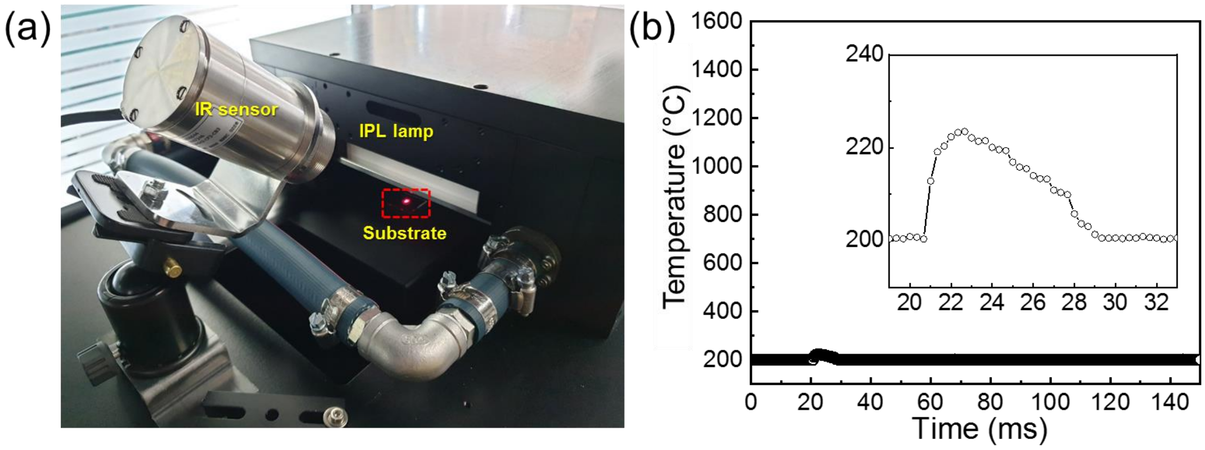

Figure S3. (a) Optical image of IR sensor system. (b) Temperature-time curve obtained as a single pulse generated at 300V with on-time of 10 ms was irradiated into the air below the lamp

For temperature measurements, samples should be put 15 cm distant from the IR sensor to locate samples in focus. Two red dots generated from the IR sensor should be brought together into one on samples (Figure S3a). Since there could be IR elements induced by IPL irradiations, temperature was measured into the air 4 cm below the lamp under various IPL conditions. Even though there are some noise elements generated as IPL flashes, it was found that effects of the IPL irradiation on measurement in temperature were negligible (Figure S3b).

At first temperatures were obtained with the emissivity value of the IR sensor set at 1.00. To identify the emissivity of GO, we measured temperature values (245 °C) on hot plate and (235 °C) on the surface of GO. Then, the emissivity of GO was calculated by the following equation

$$\frac{1}{T_{measured}} = \frac{1}{T_{corrected}} + \frac{\lambda \ln(\varepsilon_{measured}/\varepsilon_{corrected})}{C_2}$$

,where $T_{measured}$ is measured temperature values of GO when $\varepsilon_{measured}$ is set at 1.00, $T_{corrected}$ is a temperature of hot plate, λ indicates IR sensor detectable wavelength of 2.3 μm, and C_2 means second radiant constant 14,388 μm/K. As a result, $\varepsilon_{corrected}$ is estimated to be 0.79 which is analogue to the emissivity of carbon based materials such as carbon nanotubes, reduced graphene oxides, and graphite [1-4]. Therefore, we introduce 0.79 as the corrected emissivity to correct the firstly measured values with the emissivity value set at 1.00. However, the emissivity of GO will constantly be changing, depending on the reduction degree and the doping condition. The emissivity value will not be constant in any possible case, leading to a possible error in the temperature calculation. Even though the occurrence of an error is possible in the temperature calculation, it is confirmed that direct temperature

measurement by the IR sensors can give unlooked-for information (e.g. shoulder regions which are related to chemical reactions such as main reduction temperature range and doping source evaporation). In particular, it should be noted that detecting shoulder peaks is almost impossible for simulation approaches that predict temperature.

Multiple shots were used for an in-depth study of how the doping and reduction reactions progress. However, as a single shot with relatively high energy can achieve both the reduction and doping process during the irradiation time, the pulse repetition rate was not considered in depth. Despite that, it is believed that the heat accumulation effect can be utilized in various ways with multiple shots. In addition, it is noteworthy that a full charging of the capacitors in intensive pulsed light (IPL) takes longer than 1 s after light is generated via discharging the capacitors. Therefore, to induce a light source with the same energy density, capacitors should be fully charged, meaning that the interval between each pulse should be longer than 1 s. However, according to Figure 1e in the manuscript, the next pulse should be generated within 30 ms for the heat accumulation effect. Hence, the fact that each light pulse shorter than the cooling time (< 30 ms) has a different energy density should be taken into account for the multiple shot strategy

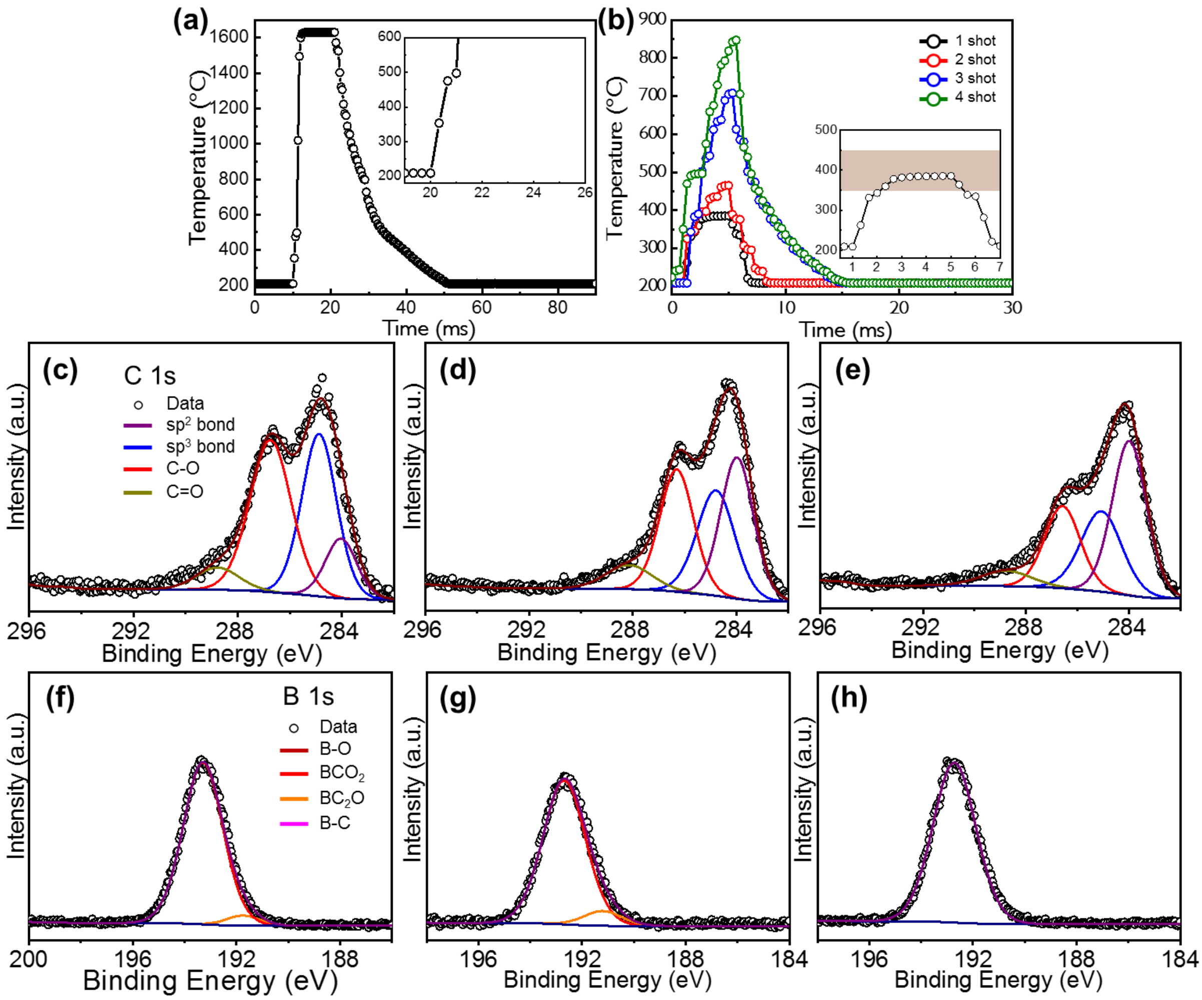

Figure S4. Temperature-time curve of (a) GO as irradiated with a single pulse of 11.0 J cm^{-2} and (b) BA@GO as irradiated with 1 to 4 shots with energy of 4.9 J cm^{-2}. *Ex-situ* XPS analysis of BA@GO on shots: High-resolution spectra in the vicinity of the C 1s for BA@GO irradiated with (c) 0 shot, (d) 2 shots, and (e) 4 shots of 4.9 J cm^{-2} and in the vicinity of the B 1s for BA@GO irradiated with (h) 0 shot, (g) 2 shots, (h) 4 shots of 4.9 J cm^{-2}, respectively. For high resolution spectra of the B 1s, BA@GO samples were rinsed at deionized (DI) water for the removal of boric acid and boric oxide residues.

As the process goes, maximum temperatures increase indicating broadening of sp^2 domain in GO sheets. Notably, it was observed from the Figure S4c–e that a peak related to C-O bonds graudally decreased to partially reduce GO sheets. Contrary to it, the high-resolution spectra of B 1s does not change, which shows that only reduction process occured without B doping in the temperature range.

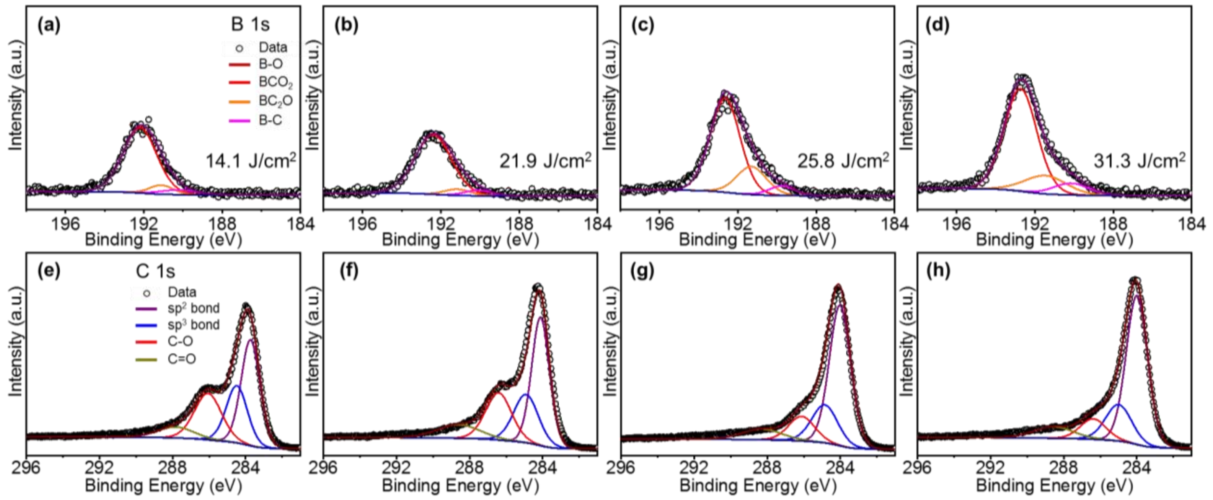

Figure S5. *Ex-situ* XPS analysis of BA@GO on shots: High-resolution spectra in the vicinity of the B 1s for BA@GO irradiated with a single shot of (a) 14.1 J cm^{-2}, (b) 21.9 J cm^{-2}, (c) 25.8 J cm^{-2}, and (d) 31.3 J cm^{-2} and in the vicinity of the C 1s for BA@GO irradiated with a single shot of (e) 14.1 J cm^{-2}, (f) 21.9 J cm^{-2}, (g) 25.8 J cm^{-2}, and (h) 31.3 J cm^{-2}, respectively.

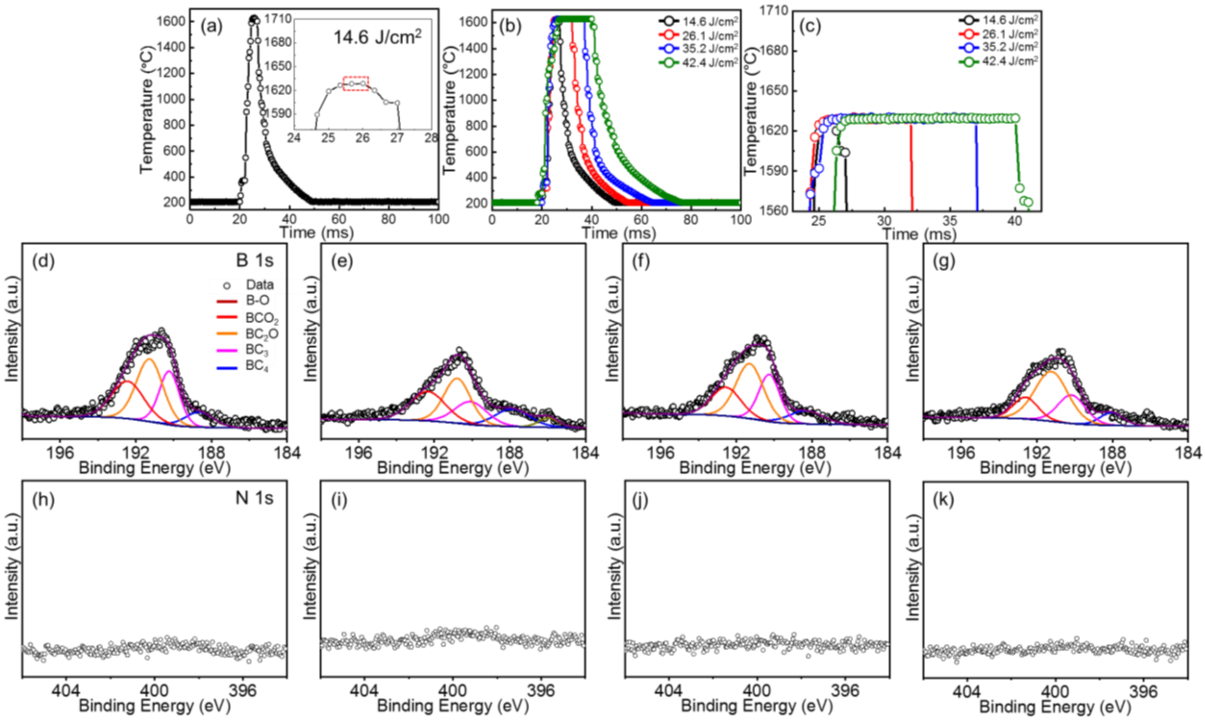

Figure S6. (a) Temperature-time curve of BA@GO obtained as a single pulse of 14.6 J cm^{-2} was irradiated. Temperature-time curve of BA@GO measured when a single pulse of 14.6 J cm^{-2}, 26.1 J cm^{-2}, 35.2 J cm^{-2}, and 42.4 J cm^{-2} was irradiated. (c) Enlarged curve of (b) in the time range from 23 to 42 ms. *Ex-situ* XPS analysis of BA@GO on shot condition: High-resolution spectra in the vicinity of the B 1s for BA@GO irradiated with a single shot of (d) 14.6 J cm^{-2}, (e) 26.1 J cm^{-2}, (f) 35.2 J cm^{-2}, and (g) 42.4 J cm^{-2} and in the vicinity of the N 1s for BA@GO irradiated with a single shot of (h) 14.6 J cm^{-2}, (i) 26.1 J cm^{-2}, (j) 35.2 J cm^{-2}, and (k) 42.4 J cm^{-2}, respectively.

Note that even though some of the light energies are estimated to be lower than the cases in Figure S5, temperature increased higher than them. It can be explained by spectrum change of light generated from the IPL equipment. The spectrum of light differs on IPL conditions. Therefore, light aborption can be enhanced, leading to higher temperature increase with lower light energy. Another possible reason is voltage drop from capacitors in the IPL equipment. Light energy is normally determined by two main factors: voltage stored in capacitors and on-time. To induce higher energy, the voltage in capacitors should be increased and longer on-time has to be set up. In this case, higher voltage and short on-time were used, leading to the dramatic voltage drop even in a short time. Therefore, it is likely that light having larger energy than estimated energy is generated from IPL.

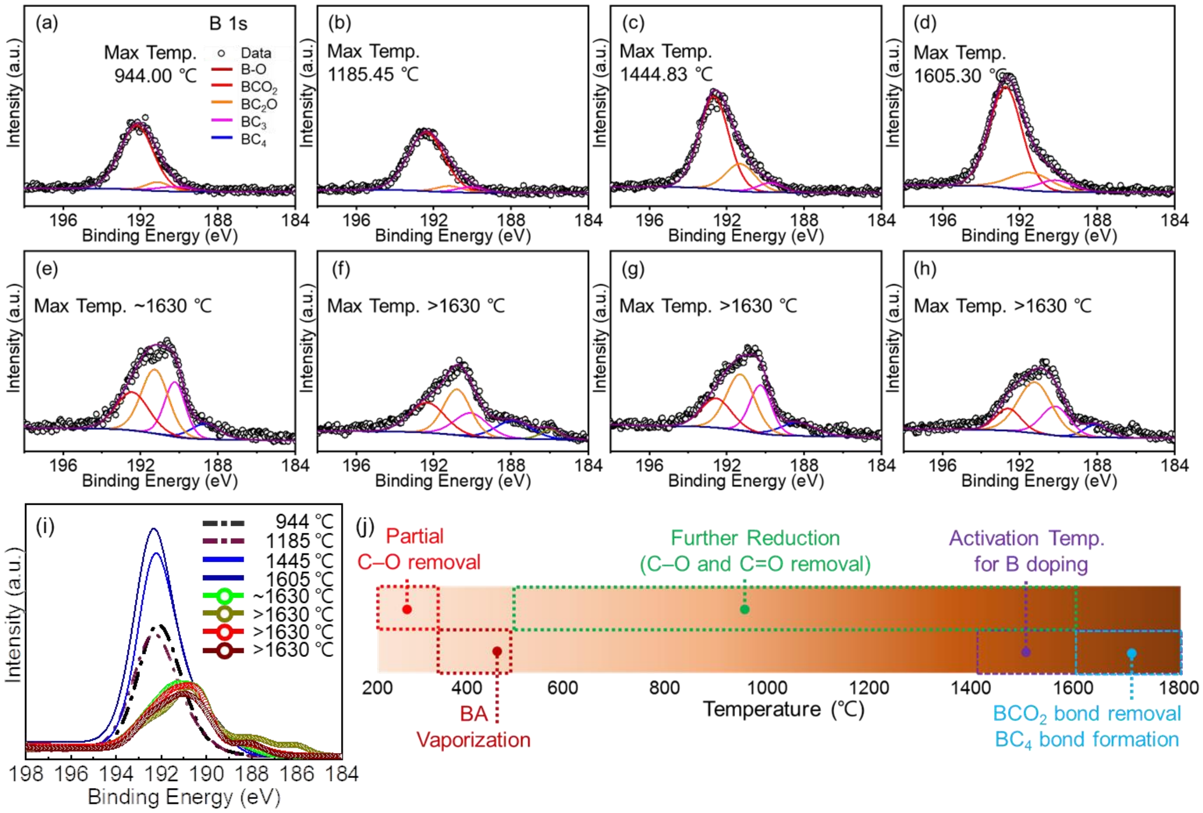

Figure S7. (a)-(h) *Ex-situ* XPS analysis: high resolution B 1s spectra of B@rGO obtained at the measured maximum temperatures by our IR sensor system with a single flash light on conditions. (i) Normalized B 1s spectra on the measured temperatures. (j) Suggested reaction mechanism on temperature through photothermal effects in millisecond scale in ambient air.

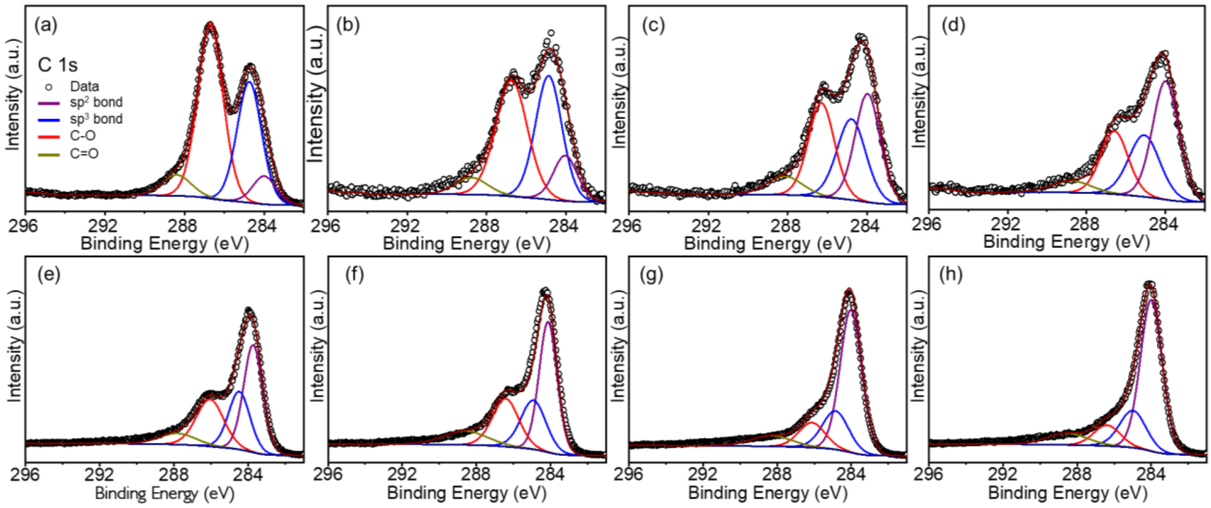

Figure S8. X-ray photoelectron spectroscopy (XPS) spectra: High-resolution spectra in the vicinity of the C 1s for (a) pristine GO, and (b) BA@GO. *Ex-situ* XPS analysis of BA@GO on shots: High-resolution spectra in the vicinity of the C 1s for BA@GO irradiated with (c) 2 shots and (d) 4 shots of 4.9 J cm^{-2}. High-resolution spectra in the vicinity of the C 1s for BA@GO irradiated with a single shot of (e) 14.1 J cm^{-2}, (f) 21.9 J cm^{-2}, (g) 25.8 J cm^{-2}, and (h) 31.3 J cm^{-2}.

From the XPS data, it was confirmed that the oxygen content could be tuned by modulating the IPL conditions. Figure S8 shows high-resolution spectra in the vicinity of the C 1s for GO, BA@GO, and B@rGO, which were subjected to photothermal treatment under various conditions. As the process proceeds, the peak intensity of the C–O bond (red line) gradually decreases, implying that stepwise reduction of GO occurs under IPL conditions. Given that the content of oxygenated functional groups, including the C–O bond, can represent their functionality, especially under the humid conditions in this study, it is believed that the oxygen content and functionality of the samples can be controlled in the proposed IPL synthesis by modulating the IPL conditions.

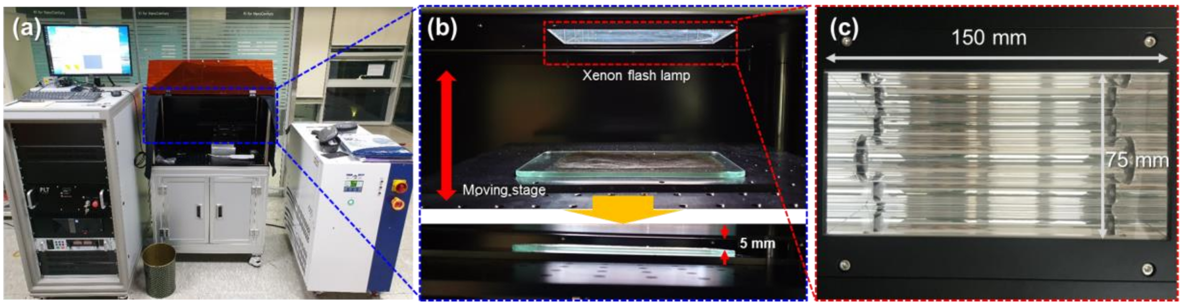

Figure S9. Optical images of (a) intensitve pulsed light system, (b) IPL treatment stage, and (c) xenon flash lamp and its reflector system.

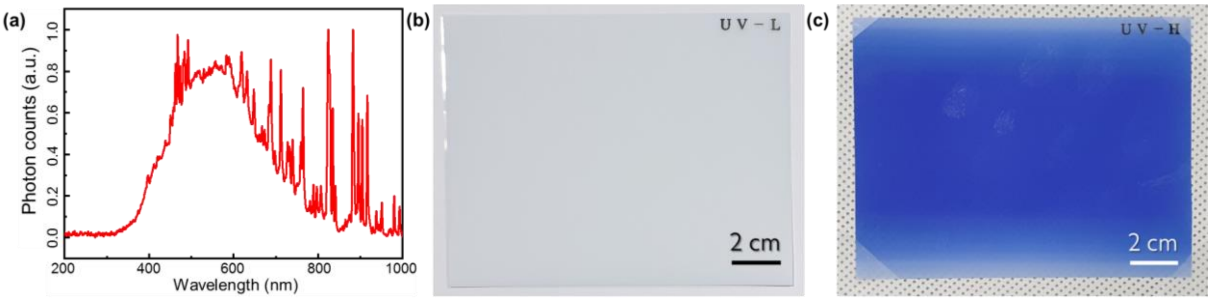

Figure S10. (a) Spectral information of xenon flash lamp. Optical images of UV sensitive papers (b) before and (c) after IPL treatment.

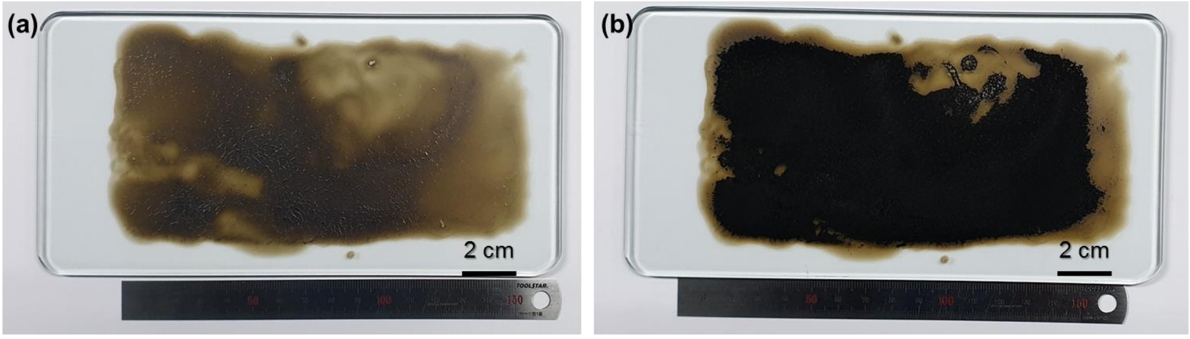

Figure S11. Photoimages (a) before and (b) after large scale synthesis of rGO with IPL.

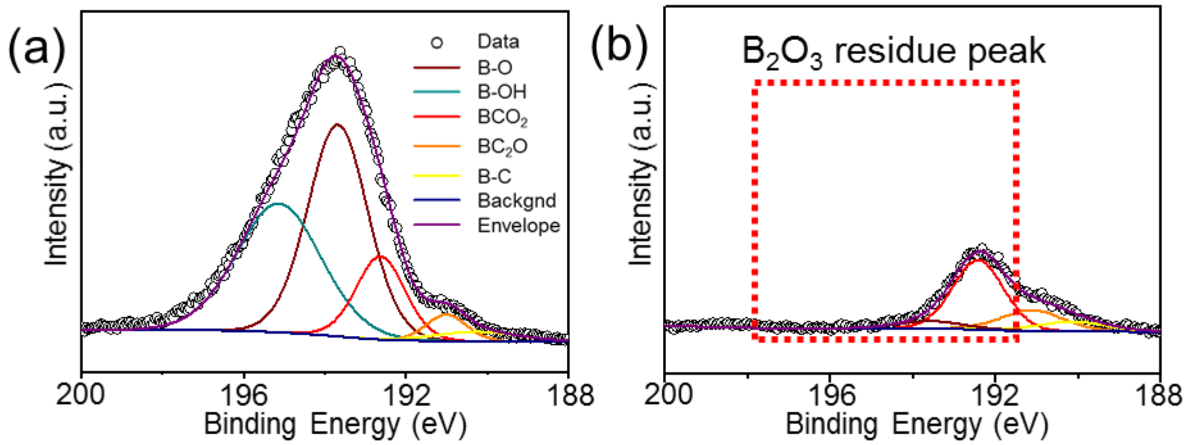

Figure S12. High-resolution XPS spectra in the vicinity of the B 1s of B@rGO (a) before and (b) after D.I. cleaning for 30 min at 60 °C.

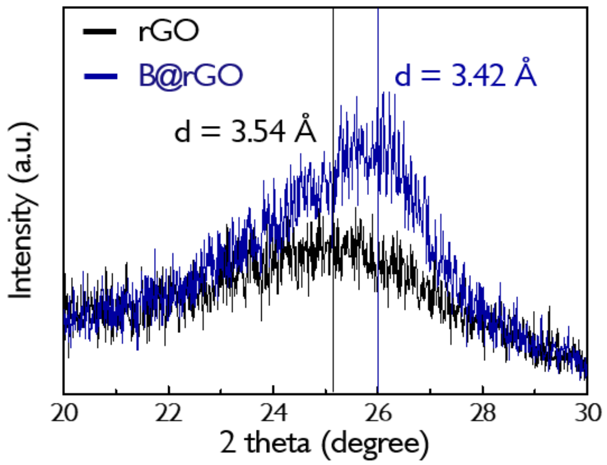

Figure S13. XRD analysis of rGO and B@rGO.

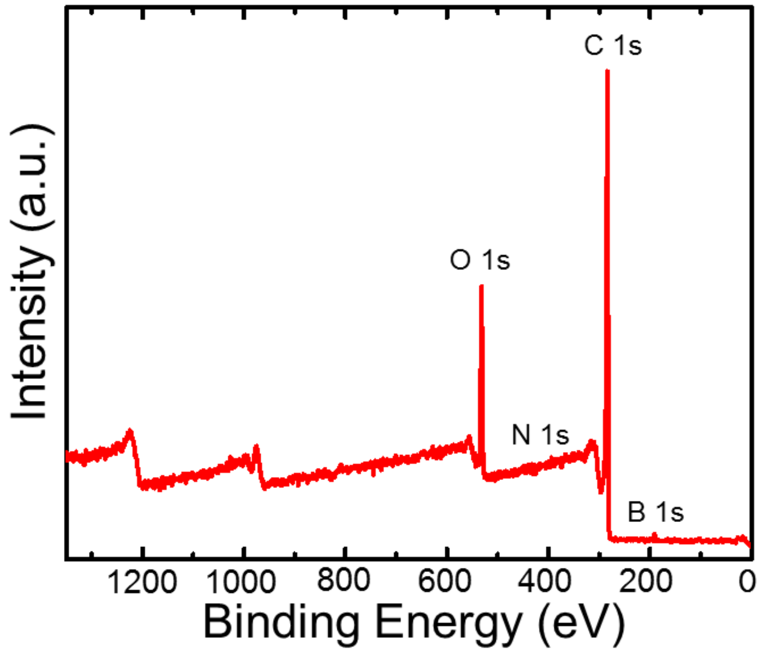

Figure S14. X-ray photoelectron spectroscopy (XPS) spectra of B@rGO.

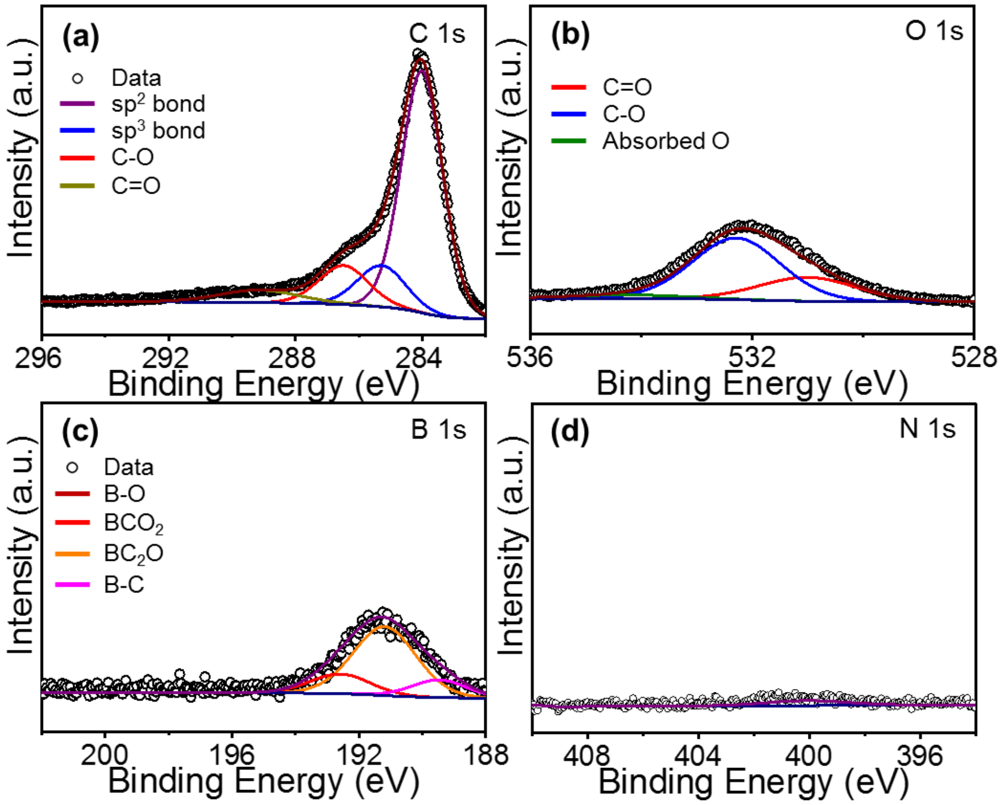

(e)

Element	C	O	B	N	Total
Atomic %	83.60	12.53	3.56	0.31	100

Figure S15. High-resolution XPS spectra in the vicinity of (a) the C 1s, (b) the O 1s, (c) the B 1s, and the N 1s for B@rGO. (e) Atomic percent of the elements of B@rGO

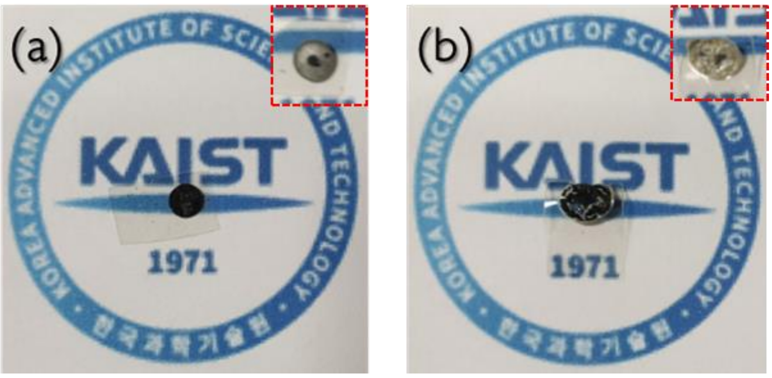

Figure S16. Photoimages of B@rGO synthesized on flexible polymer substrates (cPI) by a single irradiation with light energy of (a) 1.1 J cm^{-2} and (b) 5.6 J cm^{-2}. Inset images show the back-side of the substrates after optical heating.

Preparation of cPI film: To prepare CPI film, The PAA solution was made by dissolving 0.56 g of APS and 1.023 g of 6FDA in 3.5 g of DMF solution. After that, the mixture was stirred at 500 rpm with a magnetic stirrer for 5 h at room temperature. The uniformly dissolved PAA solution was bar-coated on glass substrates using a doctor's blade. Finally, the CPI film was obtained after imidization through a series of heat treatment at 100, 200, and 230 °C for 1 h at each temperature in a box furnace. The ramping rate was kept at 2 °C min^{-1}. After the annealing process, the CPI film formed on glass substrates was kept in DI solution for exfoliation from the glass substrates for 10 hours.

It indicates that an irradiation with energy of 5.6 J cm^{-2} induced temperature high enough to deform the substrate while the substrate treated with energy of 1.1 J cm^{-2} was not melt and wrinkled. Therefore, even though temperature rise up to 1600 °C doping and reduction even on flexible polymer substrates were achieved without damages to the substrates.

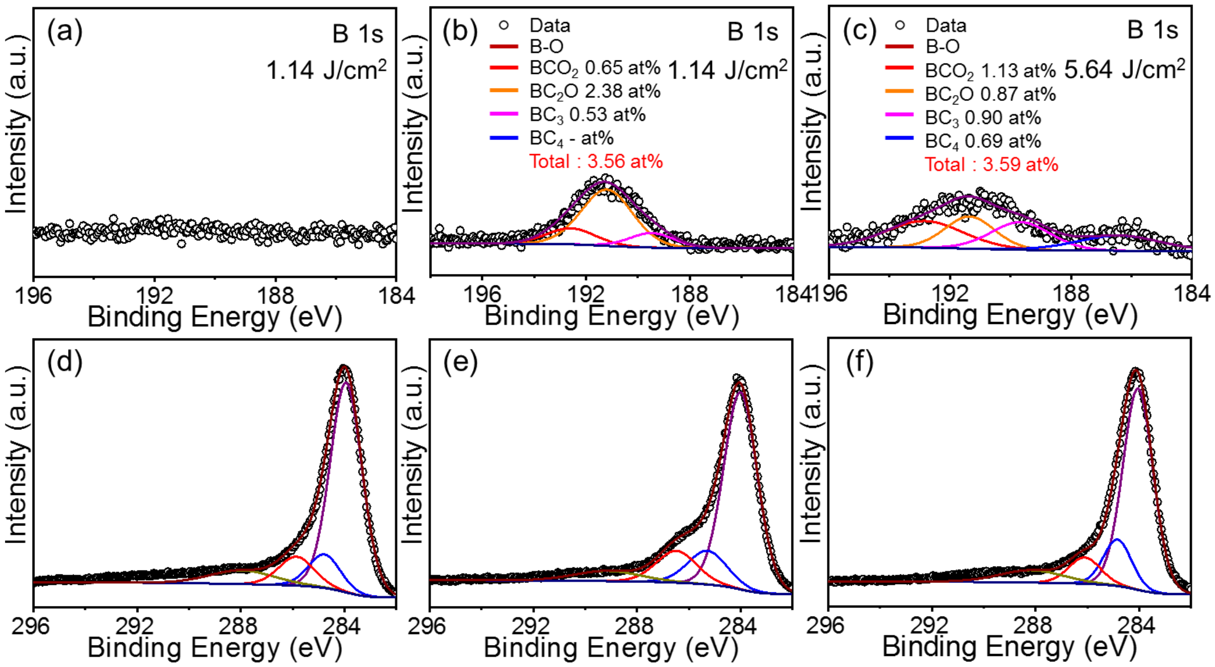

Figure S17. High-resolution XPS spectra in the vicinity of the B 1s for (a) rGO (1.1 J cm^{-2}), (b) B@rGO (1.1 J cm^{-2}), (c) B@rGO (5.6 J cm^{-2}). High-resolution XPS spectra in the vicinity of the C 1s for (d) rGO (1.1 J cm^{-2}), (e) B@rGO (1.1 J cm^{-2}), (f) B@rGO (5.6 J cm^{-2}). The energy indicates light energy generated from the IPL lamp with the large-area beam.

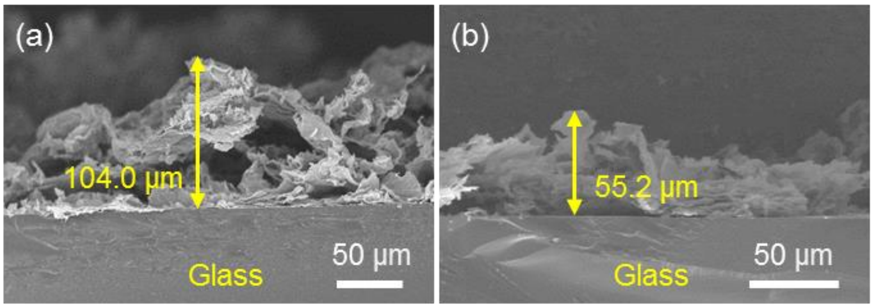

Figure S18. Cross-section SEM images of (a) rGO and (b) B@rGO.

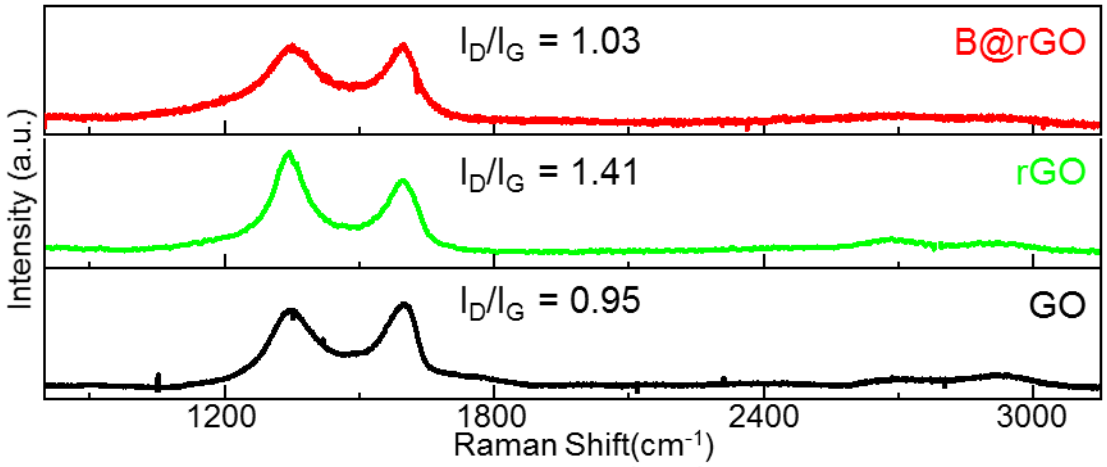

Figure S19. Raman spectra of (a) pristine GO, (b) rGO, and (c) B@rGO.

The D band peak at ca. 1350 cm^{-1} indicates the disorder and defects in graphene while the G band peak at ca. 1600 cm^{-1} relates to the carbon sp^2 bondings in the GO sheets [5]. The Raman spectrum of GO exhibits an intense D peak and G peak at 1346 and 1604 cm^{-1}, respectively. Similarly, the Raman spectrum peaks of rGO and B@rGO were found at 1344 cm^{-1} and 1350 cm^{-1} for D peak and at 1602 cm^{-1} and 1604 cm^{-1} for G peak, respectively, suggesting that no significant shifts or peak broadening was confirmed after B doping. The I_D/I_G ratio of GO (0.95), rGO (1.41) and B@rGO (1.03) was obtained to verify the degree of disorder in the materials. Notably, the I_D/I_G ratio of GO exhibits the lowest among the three. The increase of I_D/I_G ratio in rGO can arise from the decomposition of oxygen-containing functional groups, which can be ascribed to the formation of nanocrystalline sp^2 domains and the C-C cracks [6,7]. However, B@rGO showed a lower I_D/I_G value than that of rGO, implying more imperfection structure in rGO with defects. Decline in I_D/I_G value of B@rGO can be attributed to 'self-healing effects' by boron atoms on the sp^3 C defect sites in GO during the photothermal process for B doping. In turn, this leads to formation of B-C chemical bonding states [8-10].

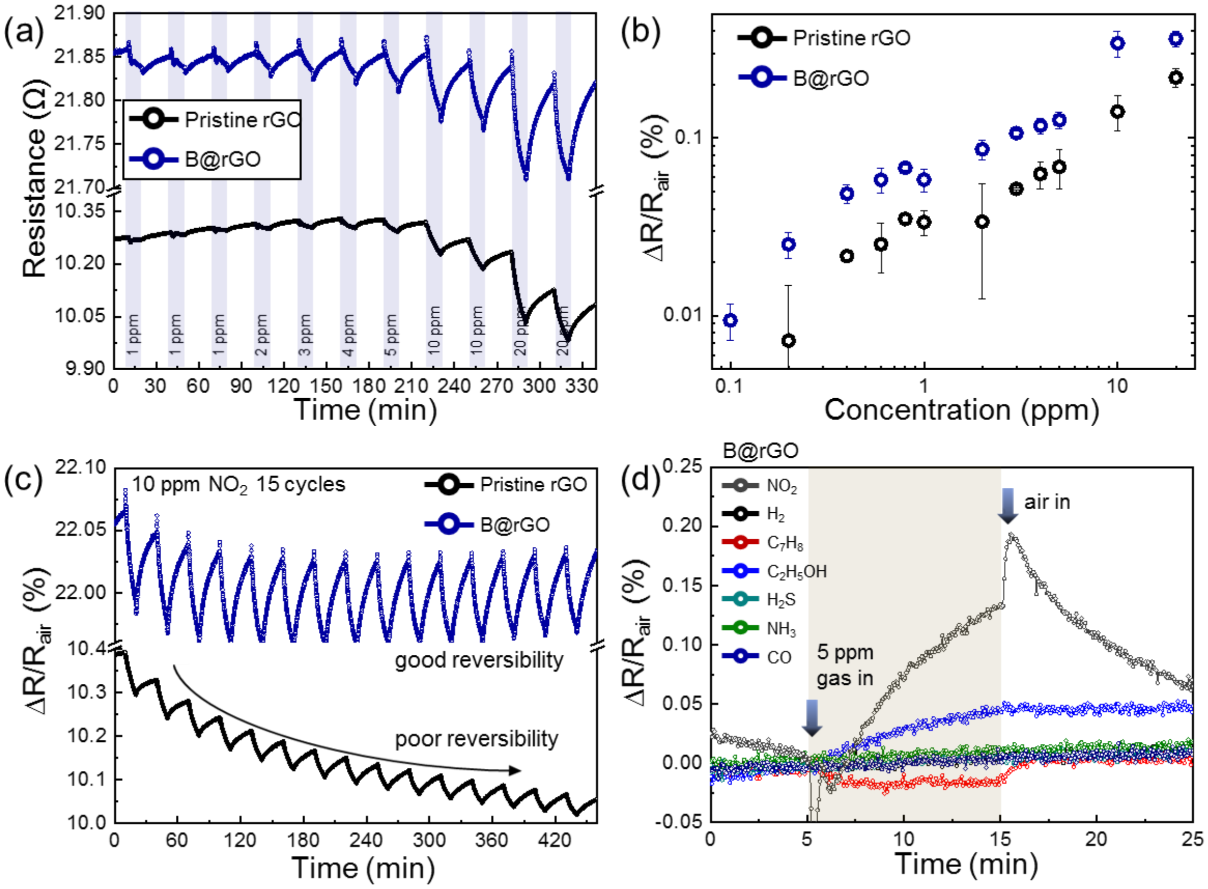

Figure S20. (a) Dynamic resistance transitions and (b) sensitivity properties of pristine rGO and B@rGO toward 1–20 ppm NO_2 concentrations. (c) Reliability of pristine rGO and B@rGO toward repetitive exposure to 10 ppm NO_2 for 15 cycles. (d) Selective detection properties of B@rGO toward 5 ppm of 7 different gas analytes. All the sensing tests were conducted in dry (1.5% RH) condition.

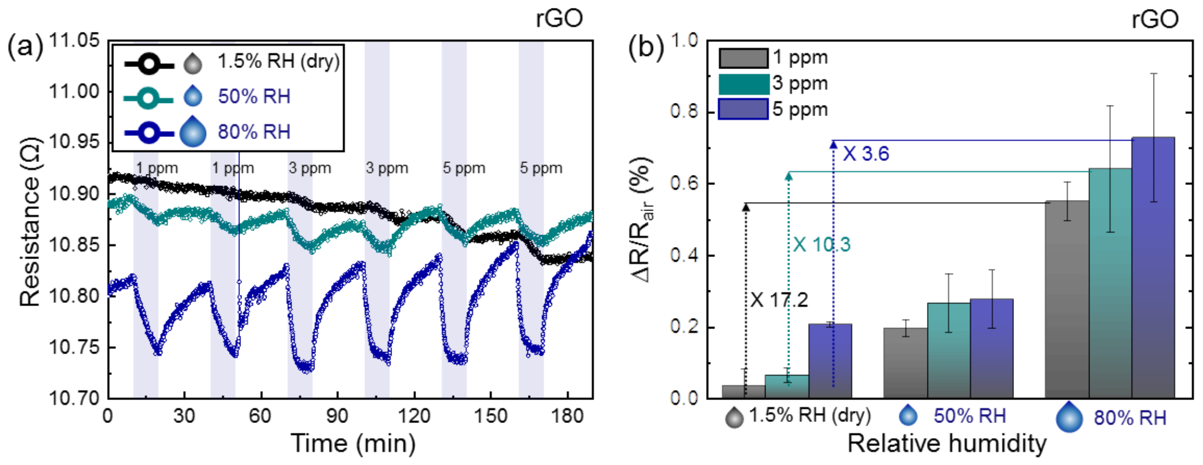

Figure S21. (a) Dynamic NO_2 sensing properties and (b) sensitivity properties of pristine rGO toward 1, 3, and 5 ppm gas concentration under three different humid conditions, that is, 1.5, 50, and 80% RH.

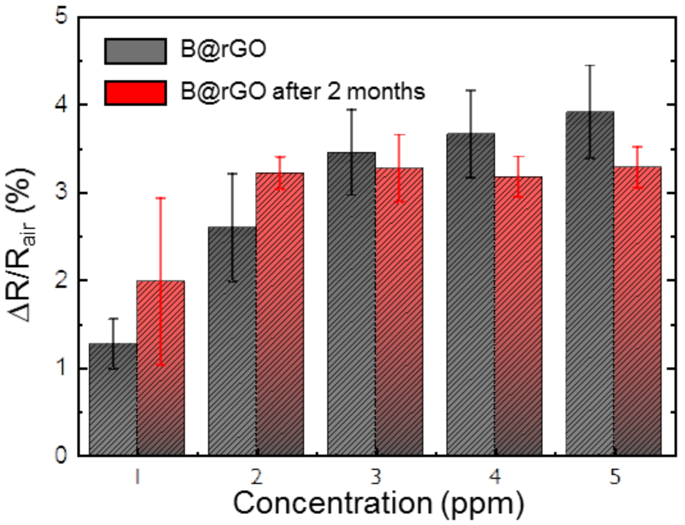

Figure S22. Long-term stability upon exposure to 1–5 ppm NO_2 using the new and after two-month B@rGO sensors.

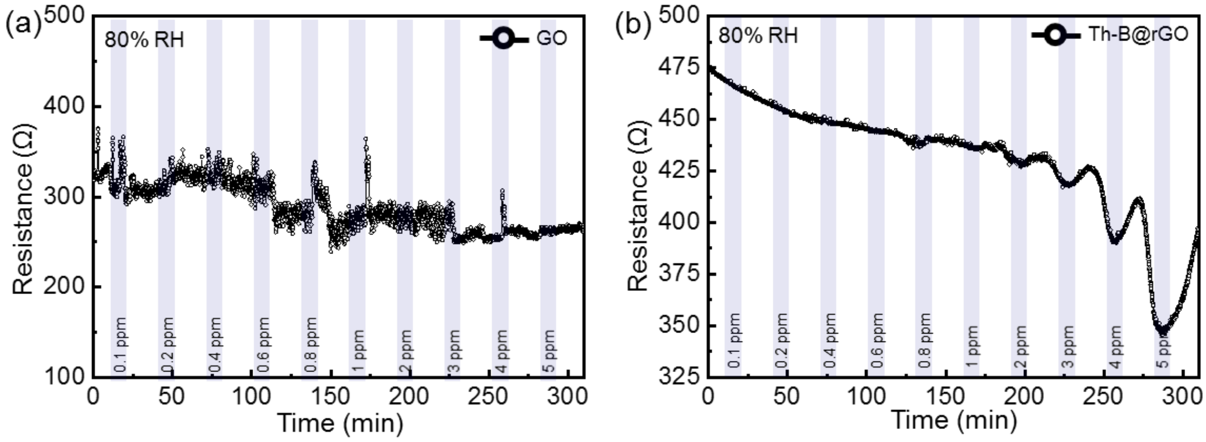

Figure S23. NO_2 sensing properties of (a) pristine GO and (b) Th-B@rGO toward 0.1–5 ppm gas concentration.

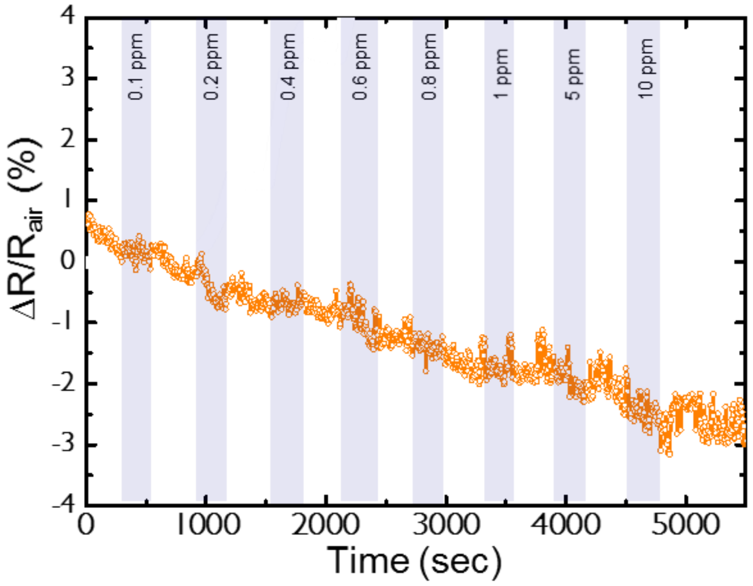

Figure S24. NO_2 sensing properties of pristine B_2O_3 toward 0.1–10 ppm gas concentration.

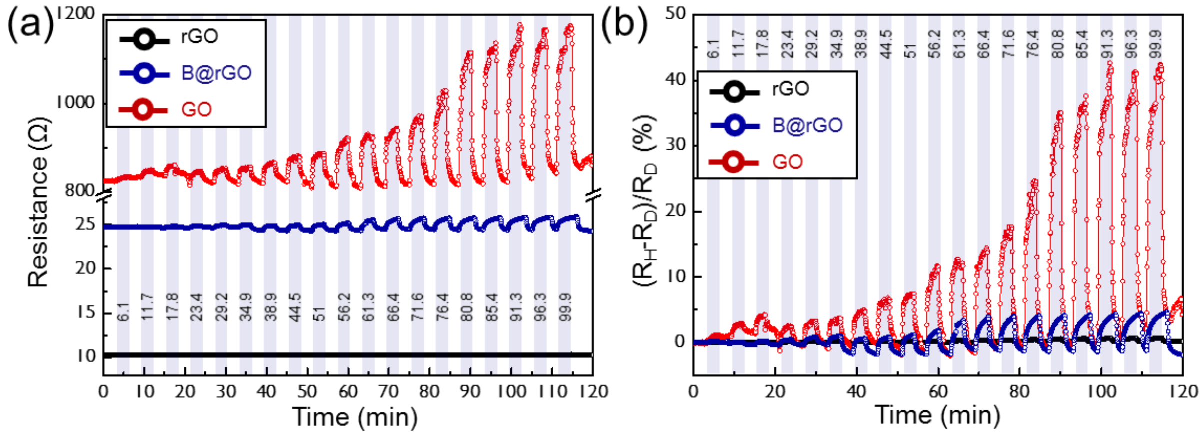

Figure S25. (a) Dynamic resistance transition and (b) dynamic sensitivity properties toward different humidity level ranging from 6.1 to 99.9% using rGO, B@rGO, and GO sensors.

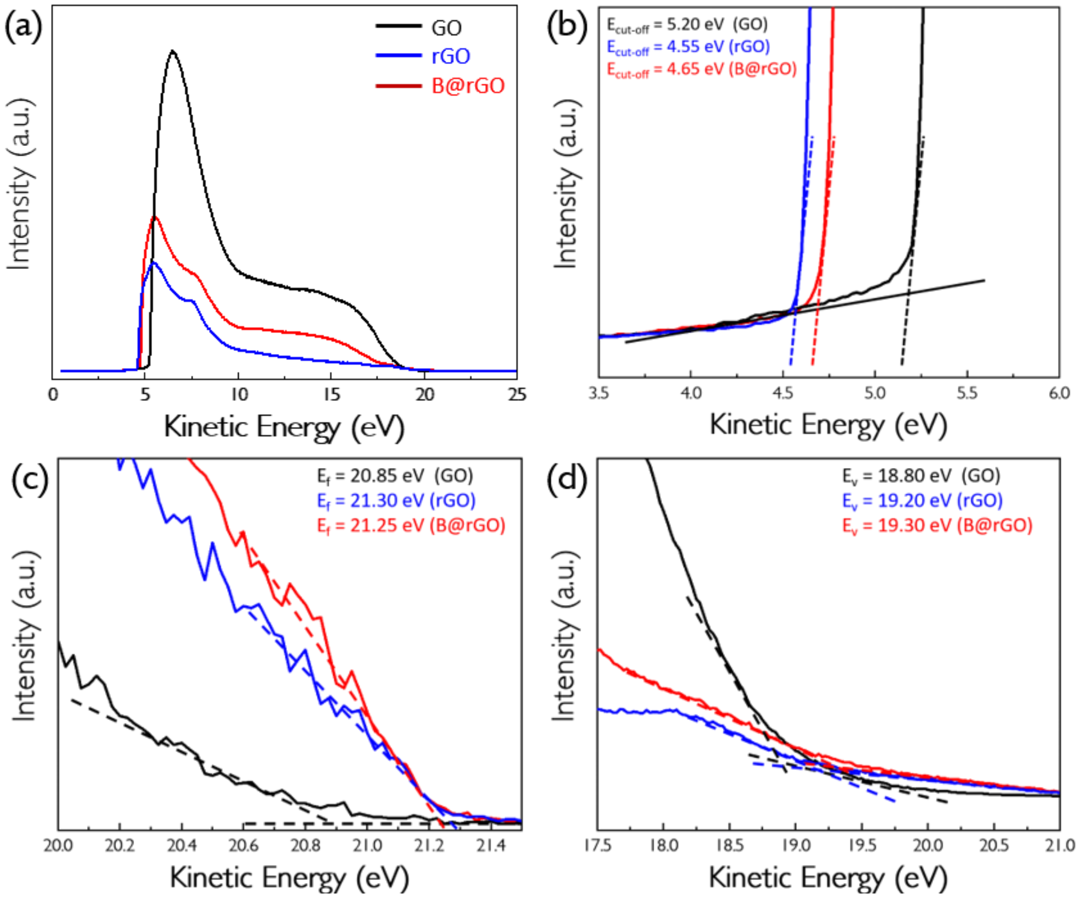

Figure S26. (a–d) UPS spectra (UV excitation by He I=21.2 eV) of GO, rGO, and B@rGO.

To investigate the work function of GO, rGO, and B@rGO, UPS analysis was conducted. The work function of the three different samples were calculated by using the following equation. Notet that the energy source in the UPS analysis is He I (21.2 eV).

$$\Phi(\text{work function}) = h\nu - |E_{cut-off} - E_f| \quad (1)$$

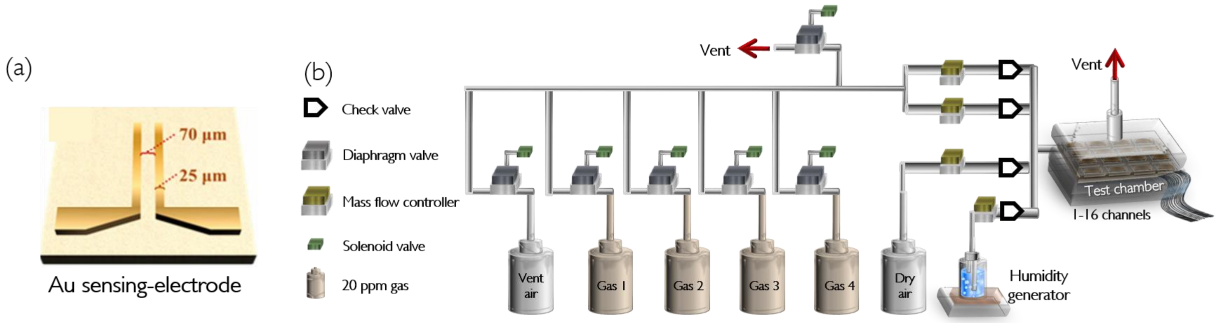

Figure S27. Schematic illustrations of the (a) alumina substrate with Au sensing-electrode and (b) gas sensor measurement system.

Table S1. Comparison with other work related to B doping into rGO.

Materials	Doping Conc.	Materials state	Doping source	Synthesis method	Application	Heating Condition	Ref.
B doped rGO	B 3.56%	Solid	Boric Acid	Optical heating	Gas sensors	A single shot (300 V) for 10 ms in air ambient	This work
B or N doped rGO	B 1.21 at% N 2.12 at%	Solid	Boric Acid	DBD plasma AC 80V, 1.6A	Supercapacitors	Plasma treatment for 3 min in H_2	11
B/N doped rGO	B ~2.5 at% N ~5 at%	Solid	Boric Acid	Ultrasonic/ Annealing	Electrocatalysts for ORR	700 °C for 1 h in N_2 (5 °C/min)	12
B doped rGO	–	Solid	Boric Acid	Annealing	Photocatalysts	300 °C for 3 h in vacuum	13
B doped rGO	B 6.2%	Frozen solid	Boric Acid	Annealing	Electrocatalytic N_2 reduction	900 °C for 3 h in H_2-Ar (5 °C/min)	14
B or N doped rGO	B 3.59 at% N 8.26 at%	Paste	Urea and boron oxide	Annealing	Photocatalysts For HER	800 °C for 2 h in Ar (10 °C/min)	15
B/N doped rGO	B 3.55 at% N 4.32 at%	Solution	$(NH_4)_2B_20_74H_20$	Microwave-Hydrothermal	Electrocatalysts for ORR	150 °C for 5 min	16
B doped rGO	–	Solution	Boric Acid	Hydrothermal	Sodium-ion Batteries	180 °C for 12 h	17
B doped rGO	–	Solid (frozen by liquid N)	Boric Acid	Annealing	Li-O_2 batteries	800 °C for 3 h in Ar (5 °C/min)	18
B doped rGO	B 1.4 at%	Solid	Boric Acid	DBD plasma AC 80V, 1.6A	Supercapacitors	Plasma treatment for 3 min in H_2	19
B doped rGO	–	Solution	Borane-tetrahydrofuran (THF) adduct	Reflux method	Photocatalysts	–	20
B/N doped rGO	B 2.38 at% N 2.66 at%	Solution	Boron tribromide(BBr_3)	Solvothermal	FET devices	in N_2	21
B doped rGO	–	Solution	Borane-tetrahydrofuran (THF) adduct	Reflux method	Supercapacitors	–	22
B doped rGO	B 3.2%	Solid	Boron oxide (B_2O_3)	Annealing	Fuel cells	1200 °C for 4 h in Ar (5 °C/min)	23

References

[1] Nat. Commun., 2015, **6**, 6834

[2] Environ. Earth Sci., 2018, **108**, 022019

[3] Appl. Surf. Sci., 2006, **253**, 778

[4] Nanotechnology, 2018, **29**, 365708

[5] Nature Nanotechnol., 2013, **8**, 235

[6] Nano Lett., 2009, **9**, 1752

[7] ACS Appl. Mater. Interfaces, 2017, **9**, 4558

[8] Chem. Commun., 2016, **52**, 10988

[9] Nanoscale, 2015, **7**, 7030

[10] J. Mater. Chem., 2012, **22**, 390

[11] J. Mater. Sci., 2019, **54**, 9632

[12] ACS Sustainable Chem. Eng., 2019, **7**, 3434

[13] J. Photochem. Photobiol. A:Chem., 2018, **364**, 130

[14] Joule, 2018, **2**, 1610

[15] ACS Appl. Mater. Interfaces, 2017, **9**, 4558

[16] Int. J. of Hydrogen Energy, 2016, **41**, 22026

[17] ACS Appl. Mater. Interfaces, 2016, **8**, 18860

[18] ACS Appl. Mater. Interfaces, 2016, **8**, 23635

[19] Chem. Commun., 2016, **52**, 10988

[20] Nanoscale, 2015, **7**, 7030

[21] Angew. Chem. Int. Ed., 2014, **53**, 2398

[22] ACS Nano, 2013, **7**, 19

[23] J. Mater. Chem., 2012, **22**, 390
